# Adolescent maturation of cortical excitation-inhibition ratio based on individualized biophysical network modeling

**DOI:** 10.1126/sciadv.adr8164

**Published:** 2025-06-04

**Authors:** Amin Saberi, Kevin J. Wischnewski, Kyesam Jung, Leon D. Lotter, H. Lina Schaare, Tobias Banaschewski, Gareth J. Barker, Arun L. W. Bokde, Sylvane Desrivières, Herta Flor, Antoine Grigis, Hugh Garavan, Penny Gowland, Andreas Heinz, Rüdiger Brühl, Jean-Luc Martinot, Marie-Laure Paillère Martinot, Eric Artiges, Frauke Nees, Dimitri Papadopoulos Orfanos, Herve Lemaitre, Luise Poustka, Sarah Hohmann, Nathalie Holz, Christian Baeuchl, Michael N. Smolka, Nilakshi Vaidya, Henrik Walter, Robert Whelan, Gunter Schumann, IMAGEN Consortium, Tomáš Paus, Juergen Dukart, Boris C. Bernhardt, Oleksandr V. Popovych, Simon B. Eickhoff, Sofie L. Valk

**Affiliations:** ^1^ Institute of Neuroscience and Medicine - Brain and Behaviour (INM-7), Research Centre Jülich, Jülich, Germany.; ^2^ Institute of Systems Neuroscience, Medical Faculty and University Hospital Düsseldorf, Heinrich Heine University Düsseldorf, Düsseldorf, Germany.; ^3^ Otto Hahn Group Cognitive Neurogenetics, Max Planck Institute for Human Cognitive and Brain Sciences, Leipzig, Germany.; ^4^ Institute of Mathematics, Faculty of Mathematics and Natural Sciences, Heinrich Heine University Düsseldorf, Düsseldorf, Germany.; ^5^ Max Planck School of Cognition, Stephanstrasse 1A, 04103 Leipzig, Germany.; ^6^ Department of Child and Adolescent Psychiatry and Psychotherapy, Central Institute of Mental Health, Medical Faculty Mannheim, Heidelberg University, Square J5, 68159 Mannheim, Germany.; ^7^ Department of Neuroimaging, Institute of Psychiatry, Psychology & Neuroscience, King’s College London, London, UK.; ^8^ Discipline of Psychiatry, School of Medicine and Trinity College Institute of Neuroscience, Trinity College Dublin, Dublin, Ireland.; ^9^ Social, Genetic and Developmental Psychiatry Centre, Institute of Psychiatry, Psychology & Neuroscience, King’s College London, London, UK.; ^10^ Institute of Cognitive and Clinical Neuroscience, Central Institute of Mental Health, Medical Faculty Mannheim, Heidelberg University, Square J5, Mannheim, Germany.; ^11^ Department of Psychology, School of Social Sciences, University of Mannheim, 68131 Mannheim, Germany.; ^12^ NeuroSpin, CEA, Université Paris-Saclay, F-91191 Gif-sur-Yvette, France.; ^13^ Departments of Psychiatry and Psychology, University of Vermont, Burlington, VT 05405, USA.; ^14^ Sir Peter Mansfield Imaging Centre School of Physics and Astronomy, University of Nottingham, University Park, Nottingham, UK.; ^15^ Department of Psychiatry and Psychotherapy CCM, Charité – Universitätsmedizin Berlin, corporate member of Freie Universität Berlin, Humboldt-Universität zu Berlin and Berlin Institute of Health, Berlin, Germany.; ^16^ German Center for Mental Health (DZPG), site Berlin-Potsdam, Berlin, Germany.; ^17^ Physikalisch-Technische Bundesanstalt (PTB), Braunschweig and Berlin, Germany.; ^18^ Institut National de la Santé et de la Recherche Médicale, INSERM U1299 “Trajectoires développementales en psychiatrie”, Université Paris-Saclay, Ecole Normale supérieure Paris-Saclay, CNRS, Centre Borelli, Gif-sur-Yvette, France.; ^19^ Department of Child and Adolescent Psychiatry, Pitié-Salpêtrière Hospital, AP-HP Sorbonne Université, Paris, France.; ^20^ Psychiatry Department, EPS Barthélémy Durand, Etampes, France.; ^21^ Institute of Medical Psychology and Medical Sociology, University Medical Center Schleswig-Holstein, Kiel University, Kiel, Germany.; ^22^ Institut des Maladies Neurodégénératives, UMR 5293, CNRS, CEA, Université de Bordeaux, 33076 Bordeaux, France.; ^23^ Department of Child and Adolescent Psychiatry, Center for Psychosocial Medicine, University Hospital Heidelberg, Heidelberg, Germany.; ^24^ Department of Psychiatry and Psychotherapy, Technische Universität Dresden, Dresden, Germany.; ^25^ Centre for Population Neuroscience and Stratified Medicine (PONS), Department of Psychiatry and Psychotherapy, Charité Universitätsmedizin Berlin, Berlin, Germany.; ^26^ School of Psychology and Global Brain Health Institute, Trinity College Dublin, Dublin, Ireland.; ^27^ Centre for Population Neuroscience and Precision Medicine (PONS), Institute for Science and Technology of Brain-inspired Intelligence (ISTBI), Fudan University, Shanghai, China.; ^28^ Department of Psychiatry, University of Cambridge, Cambridge, UK.; ^29^ German Centre for Mental Health, Berlin, Germany.; ^30^ Departments of Psychiatry and Neuroscience, Faculty of Medicine and Centre Hospitalier Universitaire Sainte-Justine, University of Montreal, Montreal, Quebec, Canada.; ^31^ Multimodal Imaging and Connectome Analysis Laboratory, McConnell Brain Imaging Centre, Montreal Neurological Institute and Hospital, McGill University, Montreal, Canada.

## Abstract

The excitation-inhibition ratio is a key functional property of cortical microcircuits which changes throughout an individual’s lifespan. Adolescence is considered a critical period for maturation of excitation-inhibition ratio. This has primarily been observed in animal studies. However, there is limited human in vivo evidence for maturation of excitation-inhibition ratio at the individual level. Here, we developed an individualized in vivo marker of regional excitation-inhibition ratio in human adolescents, estimated using large-scale simulations of biophysical network models fitted to resting-state functional imaging data from both cross-sectional (*n* = 752) and longitudinal (*n* = 149) cohorts. In both datasets, we found a widespread decrease in excitation-inhibition ratio in association areas, paralleled by an increase or lack of change in sensorimotor areas. This developmental pattern was aligned with multiscale markers of sensorimotor-association differentiation. Although our main findings were robust across alternative modeling configurations, we observed local variations, highlighting the importance of methodological choices for future studies.

## Teaser

Individualized simulations reveal a decrease in excitation-inhibition ratio in association areas throughout adolescence.

## INTRODUCTION

The vast repertoire of cortical functions emerges from a careful tuning of the interactions between excitatory and inhibitory neurons in microcircuits embedded in the structural scaffolding of the brain (*1*). Excitation and inhibition, mainly transmitted via glutamate and γ-aminobutyric acid (GABA), respectively, are inseparable and balanced, i.e., the inhibition generated in the cortical microcircuits is proportional to the local and incoming excitation (*2*). This phenomenon has been observed during both responses to external stimuli (*3*–*5*) and spontaneous cortical activity (*4*, *6*). The excitation-inhibition (E-I) balance is proposed to be essential for central aspects of cortical functioning, including the dynamic stability of activity (*7*), efficient coding of the information (*8*), sharp tuning of sensory stimuli (*2*), and generation of synchronous cortical oscillations in gamma and beta ranges (*2*, *6*, *9*, *10*). Conversely, disturbed E-I balance can lead to cortical circuit dysfunctioning and is hypothesized as a key pathophysiological mechanism under various neuropsychiatric conditions such as schizophrenia, autism spectrum disorder, and epilepsy (*11*–*15*).

Adolescence is a critical developmental period with substantial changes in the brain including maturation of the E-I ratio (*16*–*18*). During this period, several important changes occur in the architecture and function of excitatory and inhibitory neurons and synapses, which together are suggested to lead to a recalibration of the E-I ratio (*16*). For instance, postmortem histology of adolescent brains has shown a pruning of excitatory synapses within the prefrontal cortex in rats (*19*, *20*), nonhuman primates (*21*, *22*), and humans (*23*–*25*). In addition, postmortem transcriptomic studies of the prefrontal cortex in animals and humans have indicated marked changes in the expression of genes involved in inhibitory neurons and GABAergic signaling, including parvalbumin (*9*, *26*, *27*) and GABA type A (GABA_A_) receptor subunits (*28*–*31*). These transcriptomic changes are accompanied by the maturation of inhibitory function with stronger and shorter inhibitory postsynaptic currents, as observed in the prefrontal cortex of nonhuman primates (*9*, *30*), overall indicating a relative increase in inhibitory synaptic transmission in this area (*16*, *32*).

Currently available evidence on the in vivo maturation of the E-I ratio in humans is limited, as the invasive methods used in animal studies are not feasible in humans. However, in vivo proxies of the E-I ratio have been proposed, relying on its putative macroscale functional consequences captured in functional imaging (*33*, *34*) and electrophysiology (*35*–*37*) or through biochemical quantification of glutamatergic or GABAergic neurotransmitters using magnetic resonance spectroscopy (*38*–*40*). Such approaches are informative but lack a certain level of mechanistic insight and detail that is observed with, for example, direct measurement of the excitatory and inhibitory input currents as done in animal research. Furthermore, studies on the development of the E-I ratio are often focused on selected areas, primarily within the prefrontal cortex, and the knowledge on the regional patterns of E-I ratio maturation across the whole cerebral cortex is limited. Biophysical network modeling (BNM) of the brain is a promising computational technique that can bridge different scales of investigation at a whole-cortical level. It provides a tool to noninvasively derive mechanistic inferences about a hidden brain feature at the microscale, such as the E-I ratio, based on the observed (empirical) in vivo data at the macroscale and has provided valuable insights into brain (dys)function (*41*–*47*). In this approach, the dynamic spontaneous activity of brain areas is simulated using biologically realistic models that are informed by, for instance, the blood oxygen level–dependent (BOLD) signal measured during resting-state functional magnetic resonance imaging (rs-fMRI) (*48*–*51*).

In this study, we aimed to investigate the in vivo maturation of the regional E-I ratio in adolescents at an individual level. To achieve this, we applied the BNM approach on two independent cross-sectional and longitudinal neuroimaging datasets from the Philadelphia Neurodevelopmental Cohort (PNC) and the IMAGEN study (*52*, *53*). We performed large-scale simulations of individualized BNMs (*44*, *54*, *55*), in which models were informed by structural connectivity (SC) and functional imaging data of each subject. The subject-level precision of these models allowed for mapping the estimated E-I ratio specifically in each individual using simulations that best represented their empirical data and furthermore enabled studying within-subject maturation longitudinally. This extended a previous study that used the BNM approach to study E-I ratio development in the PNC dataset at the level of age groups (*56*). We demonstrated replicable effects across the two datasets, indicating cross-sectional and longitudinal age-related increases in relative inhibition in the association areas and no significant changes or relative increases in excitation in the sensorimotor areas. This pattern of the E-I ratio maturation was aligned with the proposed sensorimotor-association axis of the cortical neurodevelopment (*18*). Subsequently, given that the simulation results may be affected by various modeling and analytical choices (*57*–*59*) or might be confounded by the variability of underlying structural connectome, as well as the noise within the simulations and parameter optimization, we extensively assessed and demonstrated the robustness of our simulation-based findings against these nuisances. Last, we contrasted our marker of the E-I ratio with alternative, previously used BNM-based markers, highlighting methodological and conceptual considerations regarding their usage and interpretation.

## RESULTS

### Overview

We included 752 adolescents from the cross-sectional PNC dataset [409 female; mean age: 15.3 ± 2.4 (10 to 19) years] (*52*) and 149 participants from the longitudinal IMAGEN study (72 female; mean age: 14.4 ± 0.4 years at the baseline and 18.9 ± 0.5 years at follow-up) (*53*). Subject/session diffusion-weighted imaging (DWI) and rs-fMRI data were used to generate individual matrices of (i) structural connectome based on the density of white matter streamlines, (ii) functional connectivity (FC) matrix as the correlation of the BOLD signals, and (iii) functional connectivity dynamics (FCD) matrix as a measure of how the FC dynamically evolves through sliding windows of time during the scan across 100 cortical areas (*60*). Hereafter, we refer to FC and FCD matrices derived from the imaging data as empirical FC and FCD to distinguish them from simulated FC and FCD.

**Fig. 1.**
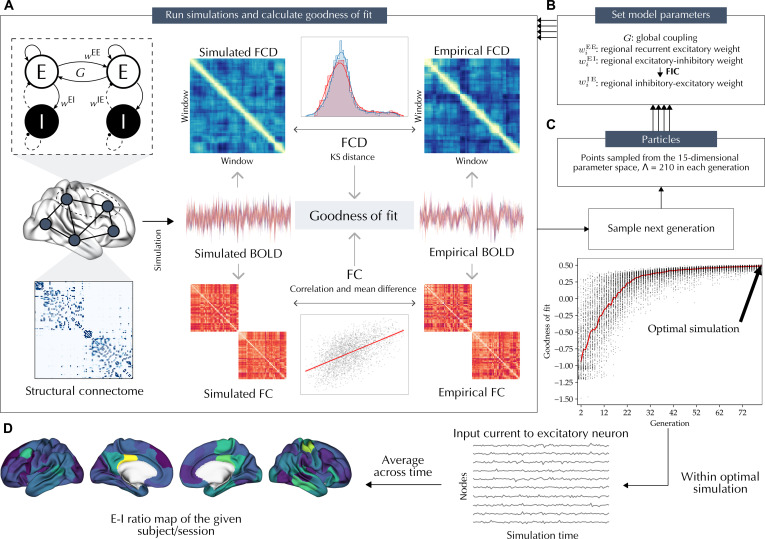
Overview. Individualized BNM simulation-optimization (**A** to **C**) was performed to derive the subject/session–specific regional measures of the E-I ratio, defined as time-averaged in silico input current to the excitatory neurons, $\left\langle \;I_{i}^{E}\;\right\rangle$ (**D**). The model consists of coupled excitatory and inhibitory neuronal pools in each node, where the excitatory neuronal pools of brain nodes are interconnected through the structural connectome of the given subject/session [(A), left]. The model is controlled by a global parameter G , which adjusts interregional coupling, in addition to regional parameters $w_{i}^{\text{EE}}$ , $w_{i}^{\text{EI}}$ , and $w_{i}^{\text{IE}}$ , which characterize the connection weights between excitatory and inhibitory neuronal pools within each node. In each simulation, G , $w_{i}^{\text{EE}}$ , and $w_{i}^{\text{EI}}$ are set by the optimizer, while $w_{i}^{\text{IE}}$ is determined on the basis of the FIC algorithm (B). The covariance matrix adaptation-evolution strategy was used to optimize model parameters given empirical data of a subject/session (C). The optimization goal was to maximize the goodness of fit by tuning 15 free parameters, including G , as well as bias and coefficient terms used to determine $w_{i}^{\text{EE}}$ and $w_{i}^{\text{EI}}$ based on six fixed biological cortical maps (fig. S1). The goodness of fit of each simulation to the empirical functional data [(A), right] was assessed as the correlation of FC matrices subtracted by their absolute mean difference and the KS distance of FCD matrices derived from the simulated or empirical BOLD signal. After completion of two optimization runs, the optimal simulation with the best goodness of fit to the empirical functional data of the target subject/session was selected (C). Last, the in silico input current to the excitatory neuron of each node $I_{i}^{E}$ was averaged across simulation time, resulting in an E-I ratio map for each subject/session (D).

Next, we performed individualized BNM simulations and parameter optimizations for each subject/session to estimate their regional measures of the E-I ratio based on their in vivo imaging data (Fig. 1). We applied the reduced Wong-Wang model (*61*), which models each node as coupled excitatory and inhibitory neuronal pools, where the excitatory neuronal pools of different nodes are interconnected through the individual-specific SC. The model was controlled by global and regional free parameters, which were fit to the empirical resting-state functional data of the target subject/session using the covariance matrix adaptation-evolution strategy (CMA-ES) optimization algorithm (*62*–*64*). This involved running a maximum of 33,600 simulations per subject/session using an efficient implementation of BNM simulations on graphical processing units (GPUs; https://cubnm.readthedocs.io). The model parameters included a global parameter G , which scales the strength of interregional coupling, in addition to regional parameters $w_{i}^{\text{EE}}$ , $w_{i}^{\text{EI}}$ , and $w_{i}^{\text{IE}}$ , which characterize the connectivity weights between excitatory and inhibitory neuronal pools within each node. Motivated by recent developments of this model (*65*, *66*), we let $w_{i}^{\text{EE}}$ and $w_{i}^{\text{EI}}$ to vary across nodes, i.e., they were computed, independent of each other, through weighted combinations of six fixed biological maps that represent microstructural, functional, transcriptomic, and neurochemical heterogeneity of the human cerebral cortex. These maps were obtained from independent healthy adult samples and included average T1-weighted/T2-weighted ratio (T1w/T2w), average cortical thickness, principal gradient of FC (FC G1), principal axis of gene expression (Gene PC1), and average *N*-methyl-d-aspartate (NMDA) and GABA type A/Bz (GABA_A/BZ_) receptor positron emission tomography maps (fig. S1) (*67*–*75*). Furthermore, in each simulation, $w_{i}^{\text{IE}}$ was determined on the basis of an analytical-numerical feedback inhibition control (FIC) algorithm, which aimed to maintain the firing rate of excitatory neurons within a biologically plausible range of 3 Hz (*61*, *66*). Then, from the optimal simulations of each subject/session, we extracted the in silico input current to the excitatory neuron of each node $I_{i}^{E}$ , averaged across simulation time, which resulted in individual-specific $\left\langle \;I_{i}^{E}\;\right\rangle$ maps. The $\left\langle \;I_{i}^{E}\;\right\rangle$ values reflect in silico estimates of the regional E-I ratio, defined as the relative level of excitation compared to the relative level of inhibition exerted onto excitatory neurons, given that $I_{i}^{E}$ results from the combination of excitatory input currents to each node (from itself and from the excitatory neurons of the other nodes through the SC) balanced by local inhibitory currents. Therefore, an increase in $\left\langle \;I_{i}^{E}\;\right\rangle$ can be interpreted as a relative increase in excitation or decrease in inhibition, i.e., an increase in E-I ratio, within a model region.

### Cross-sectional age-related variation of the E-I ratio

**Fig. 2.**
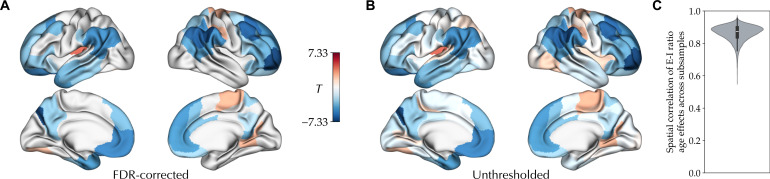
Cross-sectional effect of age on the E-I ratio during adolescence. (**A**) Effect of age on the E-I ratio, showing its significant age-related decrease (blue) and increase (red) during adolescence in the PNC dataset, after removing outliers and controlling for the goodness of fit, sex, and in-scanner rs-fMRI motion, corrected for multiple comparisons using FDR. (**B**) Unthresholded map of the effect of age on the E-I ratio. (**C**) Distribution of correlation coefficients between E-I ratio age effect maps of all pairs of subsamples across 100 half-split subsamples of the dataset.

Then, we studied cross-sectional age-related variation of the E-I ratio during adolescence in the PNC dataset. The individualized optimal simulations of the PNC dataset showed a goodness of fit of 0.259 ± 0.101 to the empirical data (fig. S2). On the basis of these simulations, we found widespread significant age-related decreases in E-I ratio in association areas within the frontal, parietal, and temporal lobes, in contrast to its age-related increases in visual and sensorimotor areas as well as the left posterior insula, controlled for goodness of fit, sex, and in-scanner rs-fMRI motion and adjusted for multiple comparisons at a false discovery rate (FDR) of 5% (Fig. 2A). The effect sizes across these regions, partial correlation of age and E-I ratio controlled for the confounds, ranged between −0.255 and 0.156. We then assessed the within-sample stability of the age effects across 100 subsamples of the data, each including half of the total sample with 376 subjects. The unthresholded age effects on the E-I ratio across all pairs of subsamples showed a mean correlation coefficient of 0.863 ± 0.047, indicating the high within-sample stability of the observed age effects (Fig. 2C). Of note, assessing maturational differences of the E-I ratio between males and females, we found no FDR-corrected significant age-by-sex interactions.

### Longitudinal changes of the E-I ratio

**Fig. 3.**
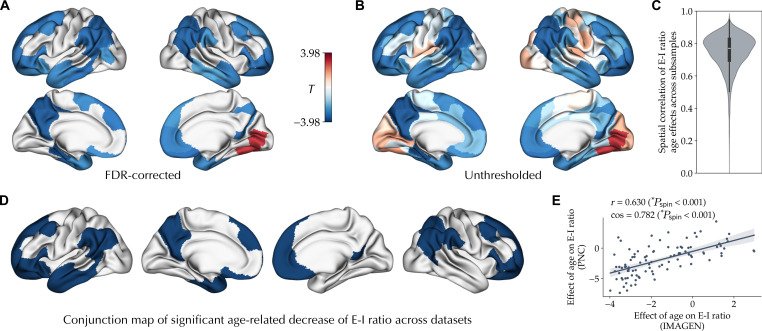
Longitudinal effect of age on the E-I ratio during adolescence. (**A**) Longitudinal effect of age on the E-I ratio, showing its significant decrease (blue) and increase (red) through adolescence, using a mixed-effects model with random intercepts for each subject, after removing outliers and controlling for goodness of fit, sex, in-scanner rs-fMRI motion, and site, corrected for multiple comparisons using FDR. (**B**) Unthresholded effect of age on the E-I ratio. (**C**) Distribution of correlation coefficients between E-I ratio age effect maps of all pairs of subsamples across 100 half-split subsamples of the dataset. (**D**) Conjunction of regions showing significant decreases in E-I ratio associated with age in the PNC and IMAGEN datasets. (**E**) Spatial coalignment [Pearson correlation (*r*) or cosine similarity (cos)] of longitudinal effects of age on the E-I ratio in IMAGEN with a cross-sectional effect of age on the E-I ratio in PNC.

To extend and assess the replicability of our findings in the cross-sectional PNC study, we next investigated the longitudinal maturation of the E-I ratio in the independent IMAGEN dataset, including 149 participants assessed at the ages of 14 and 19 years. The individualized optimal simulations in the IMAGEN had mean goodness-of-fit values of 0.266 ± 0.102 at the baseline and 0.231 ± 0.113 at the follow-up session (fig. S3). Within these simulations, we found a significant longitudinal age-related decrease in E-I ratio in widespread association areas within the frontal, parietal, and temporal lobes and a significant increase in visual areas, controlled for goodness of fit, sex, in-scanner rs-fMRI motion, and site and adjusted for multiple comparisons at an FDR of 5% (Fig. 3A). The effect sizes across these regions, calculated as the standardized mean difference of the baseline to follow-up session in E-I ratio controlled for the confounds, ranged between −0.299 and 0.229. We next assessed the within-sample stability of age effects using 100 subsamples of the IMAGEN data, each including half of the total sample with 74 subjects. This resulted in a mean correlation of *r* = 0.750 ± 0.108 between the E-I ratio age effect maps across all pairs of subsamples (Fig. 3C). Furthermore, we found no FDR-corrected significant age-by-sex interactions, yielding no evidence for sex differences in the maturation of the E-I ratio. Of note, because the quality of the tractograms in the baseline session of the IMAGEN dataset was lower, in these simulations, we used the SC of the follow-up session in the models of both sessions. However, in a subset of subjects with adequate quality of tractograms in both baseline and follow-up sessions (*n* = 110; 52 female), using models with session-specific SCs resulted in largely similar effects of age on the E-I ratio (*r* = 0.779, *P*_spin_ < 0.001; cos = 0.841, *P*_spin_ < 0.001; fig. S4).

We next assessed the similarity of cross-sectional and longitudinal age-related variation of the E-I ratio observed in the two datasets. Conjunction of regions with significant age effects on the E-I ratio in the PNC and IMAGEN datasets revealed 33 regions in the association cortices showing a significant decrease in E-I ratio, whereas no region showed a significant replicable age-related increase in E-I ratio (Fig. 3D). The mean E-I ratio across these association regions, after regressing out the effects of confounds, showed a correlation coefficient of *r* = −0.232 with age in the PNC (*T* = −6.64, *P* < 0.001) and a standardized mean difference of −0.312 between the sessions in IMAGEN (*T* = −4.17, *P* < 0.001; fig. S5). Furthermore, the unthresholded map of longitudinal effects of age on the E-I ratio in IMAGEN (Fig. 3B) was significantly coaligned (*r* = 0.630, *P*_spin_ < 0.001; cos = 0.782, *P*_spin_ < 0.001; Fig. 3E) with the map of cross-sectional effects of age on the E-I ratio observed in PNC (Fig. 2B). Therefore, overall, across the two datasets, we observed replicable cross-sectional and longitudinal effects, indicating a developmental decrease in E-I ratio in the association areas in contrast to a lack of significant changes or an increase in E-I ratio in sensorimotor areas.

### The neurodevelopmental pattern of the E-I ratio coaligns with the sensorimotor-association axis of cortical organization

**Fig. 4.**
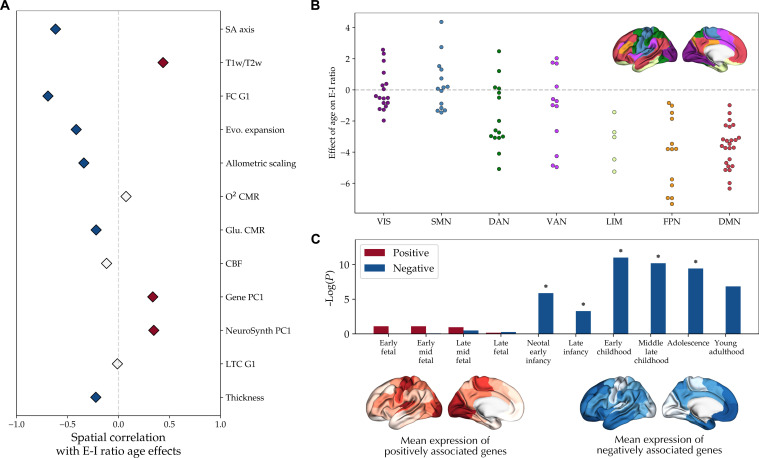
Embedding of the E-I developmental pattern in the PNC dataset along the sensorimotor-association axis. (**A**) Spatial correlation of the E-I ratio maturation map in the PNC dataset with the maps of the sensorimotor-association cortical axis based on Sydnor *et al.* (*18*) (fig. S6). Colored diamonds show statistically significant (*P*_spin_ < 0.05) positive (red) and negative (blue) spatial correlations. (**B**) Distribution of the E-I ratio maturation map across the canonical resting-state networks (*F* = 13.85, *P*_spin_ < 0.001). Post hoc tests (Bonferroni-corrected) showed significantly more positive age effects in the visual (VIS) and somatomotor (SMN) compared to the limbic (LIM), frontoparietal (FPN), and default mode networks (DMN), in addition to more positive age effects in the dorsal attention network (DAN) compared to DMN. (**C**) Bottom: Mean expression of the top 500 genes associated with the E-I ratio maturation map, split into sets of negatively associated (*n* = 187, blue) and positively associated (*n* = 313, red) genes. Top: Specific expression analysis of the two sets of genes across developmental stages in the cortex. The *y* axis shows the negative log of FDR-corrected *P* values. Asterisks denote significantly enriched developmental stages compared to null genes based on spin surrogate maps (1000 permutations) and after FDR adjustment. SA, sensorimotor association; Evo., evolutionary; CMR, cerebral metabolic rate; Glu., glucose; CBF, cerebral blood flow; NeuroSynth PC1, principal component of NeuroSynth meta-analytical maps; LTC G1, principal gradient of laminar thickness covariance.

Having observed differential effects of age on the E-I ratio across cortical areas, we next sought to investigate the embedding of this spatial neurodevelopmental pattern across different domains of cortical organization as well as developmental transcriptomics. We first studied the spatial coalignment of the maps of E-I ratio maturation with a previously proposed sensorimotor-association axis of cortical neurodevelopment and the multimodal cortical features it was composed of (fig. S6 and table S1) (*18*). The maps of E-I maturation observed in both datasets were significantly (*P*_spin_ < 0.05) correlated with the sensorimotor-association axis map (PNC: *r* = −0.617; IMAGEN: *r* = −0.607) as well as several of its components, notably including FC G1 (PNC: *r* = −0.691; IMAGEN: *r* = −0.641) and T1w/T2w (PNC: *r* = 0.437; IMAGEN: *r* = 0.548; Fig. 4A and fig. S7A). Next, comparing the maps of E-I maturation across seven canonical resting-state networks (*76*), in both datasets, we observed more negative age effects in the default mode, limbic and frontoparietal networks compared to the somatomotor and visual networks (Fig. 4B and fig. S7B). These findings indicated the coalignment of the E-I ratio maturational pattern with the sensorimotor-association axis of the cortex with a higher age-related relative increase in inhibition toward the association areas.

Last, we performed developmental transcriptomics enrichment analysis of the E-I ratio maturation maps. Using partial least squares regression with the gene expression maps obtained from the Allen Human Brain Atlas (*71*, *73*), we identified the top 500 genes expressed higher toward the negative and positive ends of the E-I ratio maturation maps. Next, we investigated the developmental enrichment of the two sets of genes using specific expression analysis of the BrainSpan dataset (*77*), comparing them against null sets of genes expressed in alignment with spun surrogates of the E-I ratio maturation maps (1000 permutations). We found the genes expressed toward the negative ends of the E-I ratio maturation maps to be enriched in later stages of development, significantly during neonatal to adolescence (PNC) or early childhood (IMAGEN) stages, in contrast to the genes expressed toward the positive ends of the E-I ratio maturation maps that were enriched in earlier fetal stages of development, although not significantly (Fig. 4D and fig. S7D).

### Sensitivity analyses

**Fig. 5.**
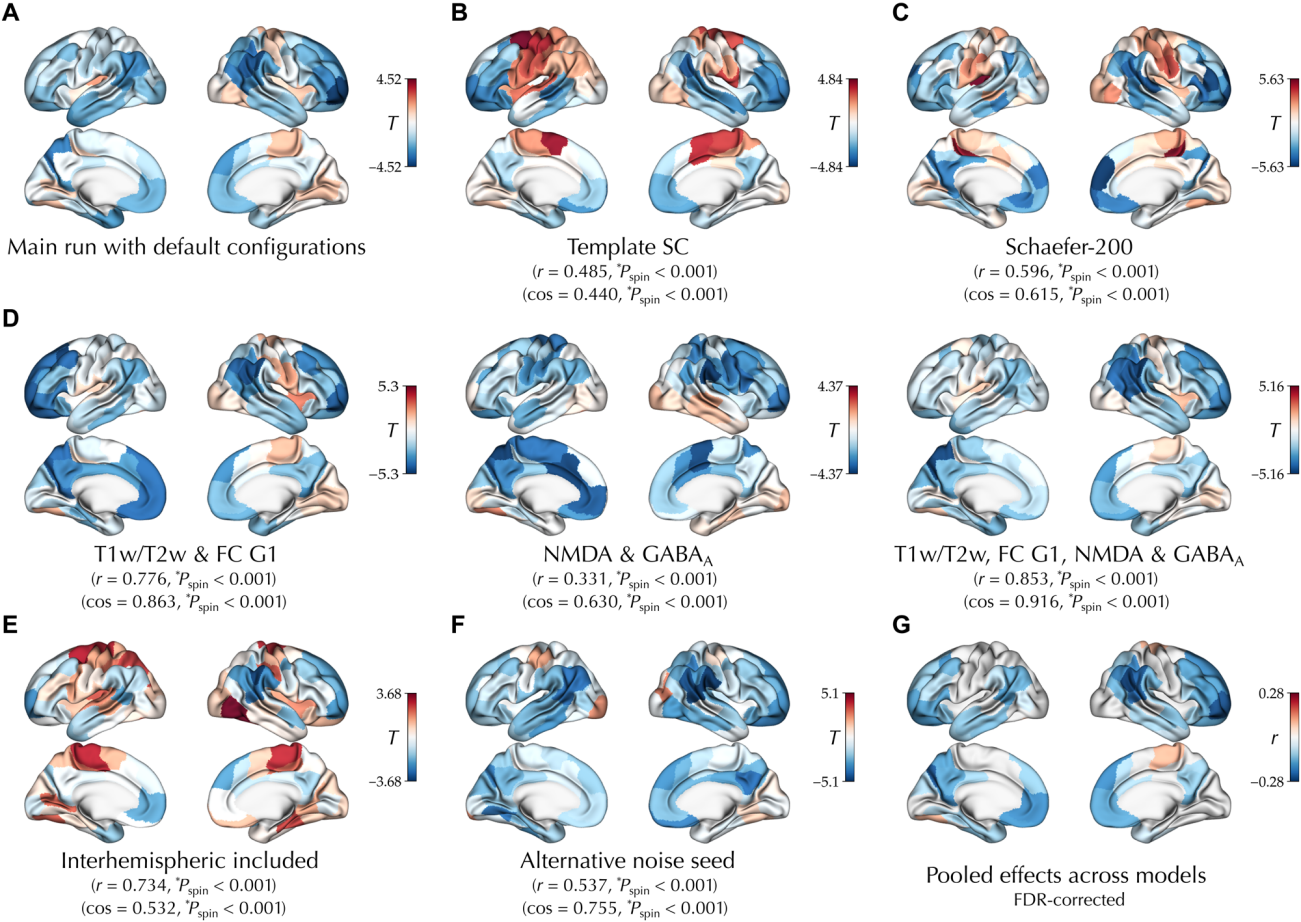
Sensitivity analyses. The unthresholded effect of age on the E-I ratio observed in a random subsample of the PNC dataset (*n* = 200) using the default configurations (**A**) compared to age effects observed using alternative configurations, including the following: (**B**) using a fixed template SC based on the MICs dataset, (**C**) definition of nodes based on a Schaefer parcellation with higher granularity of 200 nodes, (**D**) using alternative subsets of biological maps to determine the heterogeneity of regional parameters, (**E**) including the interhemispheric connections in the goodness of fit, and (**F**) using an alternative Gaussian noise seed. In (B) to (F), the statistics indicate spatial coalignment [Pearson correlation (*r*) or cosine similarity (cos)] of each map with the E-I ratio maturation map observed using the default configurations (A). (**G**) Pooled partial correlation of age with the E-I ratio (controlled for goodness of fit, sex, and in-scanner rs-fMRI motion) across (A) to (F) based on random-effects meta-analyses.

Thus far, we observed consistent age effects in the adolescent maturation of the E-I ratio in two independent cross-sectional and longitudinal datasets across a sensorimotor-association axis by using simulations of individualized BNMs. However, these simulation-based findings may be sensitive to various modeling and analytical choices (*57*–*59*) as well as confounding effects of the underlying structural connectome or noise. Therefore, we next assessed the sensitivity of the E-I ratio and its age-related changes to such nuisances, including the effects of the interindividual variability of SC, modeling configurations, and the randomness within the optimizer and the simulations. To reduce the computational costs, we limited these analyses to a random subsample of 200 subjects from the PNC dataset. Hence, the effect of age on the E-I ratio in the “main run” was recalculated in this subsample for the comparisons with the alternative runs (Fig. 5A).

#### Interindividual variability of structural connectome

Using subject-specific SCs in the main analyses enabled modeling of brain function within an individualized structural scaffold, which better represents each subject. However, this potentially introduces interindividual variability of SCs as a source of variability in E-I ratio, particularly given that $I_{i}^{E}$ is directly related to the SC in the model Eq. 1. As a result, the associations of age with model-derived features may be confounded by age-related variation of trivial features of SC, such as the node-wise strength. However, the effect of age on the E-I ratio was robust to controlling for the node-wise SC strength as an additional confound (*r* = 0.960, *P*_spin_ < 0.001; cos = 0.977, *P*_spin_ < 0.001; fig. S8A). Furthermore, when SC variability was eliminated by using an identical template SC in the BNM simulations of all the subjects, the effect of age on the E-I ratio was coaligned with the effects observed in the main run (*r* = 0.485, *P*_spin_ < 0.001; cos = 0.440, *P*_spin_ < 0.001; Fig. 5B and fig. S8B) despite a poor average test-retest reliability of E-I ratio compared to the main run {intraclass correlation coefficient (ICC): 0.319 ± 0.165; range: [−0.179, 0.632]; fig. S8B}.

#### Parcellation

Using an alternative Schaefer parcellation with higher granularity of 200 nodes (*60*), the effect of age on the E-I ratio was largely consistent with the main run based on 100 nodes (*r* = 0.596, *P*_spin_ < 0.001; cos = 0.615, *P*_spin_ < 0.001; Fig. 5C and fig. S9A), yet we observed the poor average test-retest reliability of E-I ratio compared to the main run {ICC: 0.356 ± 0.186; range: [−0.075, 0.836]; fig. S9B}.

#### Heterogeneity of regional parameters

We found consistent effects of age on the E-I ratio when alternative subsets of the six biological maps were used to determine the heterogeneity of regional parameters $w_{i}^{\text{EE}}$ and $w_{i}^{\text{EI}}$ (Fig. 5D and fig. S10): (i) using only T1w/T2w and FC G1 maps (*r* = 0.776, *P*_spin_ < 0.001; cos = 0.863, *P*_spin_ < 0.001), (ii) using only NMDA and GABA_A/BZ_ maps (*r* = 0.331, *P*_spin_ < 0.001; cos = 0.630, *P*_spin_ < 0.001), and (iii) using T1w/T2w, FC G1, NMDA, and GABA_A/BZ_ maps (*r* = 0.853, *P*_spin_ < 0.001; cos = 0.916, *P*_spin_ < 0.001). Notably, the average ICC of E-I ratio compared to the main run was respectively 0.698 ± 0.108 {range: [0.183, 0.881]}, 0.530 ± 0.133 {range: [0.203, 0.795]}, and 0.819 ± 0.068 {range: [0.483, 0.931]}, indicating its moderate to good average test-retest reliability when using alternative sets of maps.

Next, we asked to what extent the spatial pattern of E-I ratio maturation is influenced by the spatial pattern of the underlying heterogeneity maps. To investigate this, we performed an alternative run by using a set of six null maps, generated by random spinning of the six original maps on the cortical surface. In this null run, we observed weak and significant correlation of the E-I maturation map with two of the six null maps but none of the original maps. Conversely, the E-I maturation map of the main run based on the original maps was correlated more strongly with five of six original maps but only one of the null maps. This indicates that the choice of heterogeneity maps can to some extent influence the findings, although the nature of this influence is not trivial (fig. S11A).

This raises the question of whether one could use map-free alternative models that are free of this influence. To address this, we tested two map-free models including (i) a “homogeneous” model (three free parameters), which assumes the homogeneity of regional parameters and led to a significant drop of the goodness of fit by an average of −0.113 ± 0.062 (fig. S11B), and (ii) a “node-based heterogeneous” model (201 free parameters), which allows regional parameters of the nodes to vary independently as separate free parameters. Using the same number of simulations as the main run to fit this model, we observed a significant drop of the goodness of fit by an average of −0.059 ± 0.069 (fig. S11B), likely due to a reduced rate of optimizer convergence (fig. S11C). This indicates the need for a much larger number of simulations to fit this complex model, which would be infeasible in our current, subject-level BNM approach. Thus, we argue that although these map-free alternatives can avoid the influence of maps, they either are too simplistic and lack enough biological detail (“homogeneous”) or are too complex and difficult to fit at this scale (“node-based heterogeneous”), both leading to a worse model fit. This in turn highlights the necessity of an intermediate feasible solution which is to induce the constrained heterogeneity of the regional parameters based on a set of biological maps. The effect of age on the E-I ratio based on these map-free models is reported in fig. S11D for the interested reader, although we refrain from interpreting these findings given the aforementioned issues of these models.

#### Inclusion of interhemispheric connections in the cost function

When interhemispheric connections were considered in the cost function, the effect of age on the E-I ratio was consistent with the main run (*r* = 0.734, *P*_spin_ < 0.001; cos = 0.532, *P*_spin_ < 0.001; Fig. 5E and fig. S12), yet the E-I ratio showed poor average test-retest reliability compared to the main run {ICC: 0.484 ± 0.128; range: [0.071, 0.743]}.

#### Conduction velocity

Next, we assessed the potential impact of interregional conduction delay at the individual level by repeating the optimal simulations while adding conduction delay between model regions informed by the subject-specific tractograms. We calculated the ICC of E-I ratio between optimal simulations with and without conduction delay and found its good test-retest reliability with a mean of 0.977 ± 0.017 using a velocity of 1 m/s to 0.997 ± 0.002 using a velocity of 6 m/s (fig. S13).

#### Optimization random seed

Within the parameter space, multiple local optima could exist that feature different E-I ratio values. To assess this, we calculated the node-wise ICC of E-I ratio across the optimal points obtained from the two CMA-ES runs of each subject. We found a mean ICC of 0.946 ± 0.021 {range: [0.874 to 0.982]} across nodes, indicating the good test-retest reliability of E-I ratio across alternative optima (fig. S14). Furthermore, in a ground truth recovery analysis, we fitted the model to synthetic functional data with known parameters and regional E-I ratio values. The recovered E-I ratio values showed a Pearson correlation of 0.977 (*P* < 0.001) and an ICC of 0.948 with the ground truth (fig. S15). Together, these findings indicate a low risk of optimizers’ convergence to different local optima with respect to the E-I ratio.

#### Gaussian noise random sequence

We next investigated the impact of the random sequence (seed) of Gaussian noise introduced into the simulations by performing three analyses:

1) Ground truth recovery with different noise seeds: When using a different noise random seed than the one used in the ground truth simulation, the recovery fits decreased (0.811 ± 0.018, across 50 alternative seeds), with a lower Pearson correlation (0.672 ± 0.132) and ICC (0.339 ± 0.150) between the recovered and ground truth E-I ratio values, compared to when the same noise seed was used (fig. S15).

2) Test-retest reliability of E-I ratio in the optimal simulations across different noise seeds: We repeated the optimal simulations of each subject obtained in the main run and based on the default noise seed, with 50 alternative noise seeds. The median node-wise ICC of E-I ratio between the simulations using the original versus alternative noise seeds had an average of 0.810 ± 0.100 {range: [0.368 to 0.929]} across regions (fig. S16).

3) Comparing optimization runs performed using the default versus alternative noise seed: Running the full simulation-optimization process with a different Gaussian noise random sequence than the main run, we observed the poor average test-retest reliability of regional E-I ratio estimates {ICC: 0.471 ± 0.172; range: [−0.149, 0.771]}, indicating a notable effect of noise sequence on individual estimates. Nevertheless, the age-related E-I ratio effects remained largely consistent (*r* = 0.537, *P*_spin_ < 0.001; cos = 0.755, *P*_spin_ < 0.001; Fig. 5F and fig. S17). Furthermore, when age effects were assessed on the basis of the average E-I ratio values across the two runs using the default and alternative random seeds (fig. S17D), they remained highly consistent with the effects observed in either run separately (*r* = 0.844, *P*_spin_ < 0.001; cos = 0.912, *P*_spin_ < 0.001 for the main run; *r* = 0.854, *P*_spin_ < 0.001; cos = 0.930, *P*_spin_ < 0.001 for the alternative run).

Together, these findings suggest that while the Gaussian noise random sequence influences simulation outcomes, the observed effects of age on the E-I ratio remain largely robust to this influence.

#### Most consistent effects of age on the E-I ratio across alternative modeling configurations

Given the variability of the effects of age on the E-I ratio across the alternative models described above (Fig. 5, A to F), we next aimed to reconcile these findings and identify the most consistent effects of age on the E-I ratio across the different models. We performed random-effects meta-analyses, at the level of each parcel, to pool the partial correlation of age with the E-I ratio observed across the main and alternative configurations. We found a significant pooled age-related decrease in E-I ratio in association areas in frontal, parietal, and temporal lobes and its pooled increase in sensorimotor areas (Fig. 5G). The heterogeneity of the observed effects differed across the cortex and was higher in the somatomotor and parietal regions compared to visual and frontal regions (fig. S18A).

### Alternative BNM-based measures of the E-I ratio

In vivo estimation of the E-I ratio based on BNMs has been the aim of several previous studies using this or similar models (*47*, *56*, *78*–*80*). Yet, there has been no consensus on the BNM-based measures of the E-I ratio and various measures have been proposed and used across studies. Here, we present our findings regarding alternative BNM-based measures of the E-I ratio used in the previous literature and highlight some considerations regarding their usage.

**Fig. 6.**
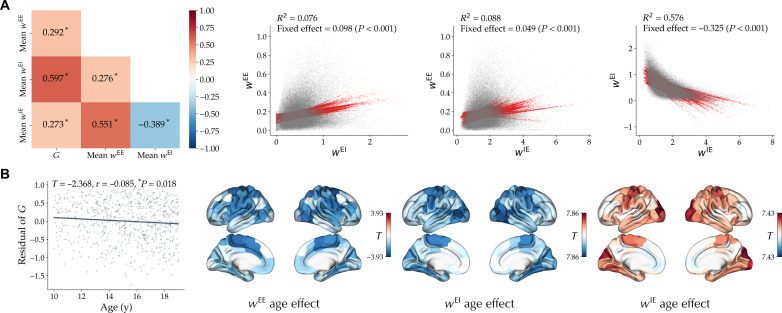
Optimal model parameter interrelation and association with age in the PNC dataset. (**A**) Left: Pearson correlation of model parameter G and brain-averaged values of regional parameters $w^{\text{EE}}$ , $w^{\text{EI}}$ , and $w^{\text{IE}}$ across subjects are shown. Asterisks denote statistically significant correlations. Right: Interrelation of regional values of parameters $w^{\text{EE}}$ , $w^{\text{EI}}$ , and $w^{\text{IE}}$ across nodes and subjects based on a linear mixed-effects model with random intercepts and slopes per each node. (**B**) Left: Effect of age on optimal parameter G . Points represent the residual of G for each subject after removing confounds. Right: Unthresholded effect of age on regional parameters $w^{\text{EE}}$ , $w^{\text{EI}}$ , and $w^{\text{IE}}$ . Age in years (y)

First, the optimal model parameters have been commonly used as measures of the E-I ratio (*47*, *78*–*80*). Variation of these parameters can be interpreted as a shift of the ratio toward higher excitation (e.g., increase in G or $w^{\text{EE}}$ ) or higher inhibition (e.g., increase in $w^{\text{EI}}$ or $w^{\text{IE}}$ ). However, in a multidimensional model in which these parameters can simultaneously covary and may be degenerate, the interpretation of their variations is not straightforward. Across optimal simulations of subjects in the PNC dataset, we found significant associations between optimal parameters, such as a negative association of $w^{\text{EI}}$ and $w^{\text{IE}}$ , indicating that lower excitatory-to-inhibitory connection weights are accompanied by (compensatory) higher inhibitory-to-excitatory connection weights (Fig. 6A). These associations were also reflected in the effects of age on the parameters (Fig. 6B). For example, there was an inverse correlation between the unthresholded effects of age on $w_{i}^{\text{EI}}$ and $w_{i}^{\text{IE}}$ (*r* = −0.706, *P*_spin_ < 0.001). The observed covariance between model parameters and their age effects indicates that these age effects should not be interpreted in isolation, and on the basis of these data, the net effect of age on the E-I ratio remains ambiguous.

In our study, as a solution to this problem of degeneracy between model parameters, we focused on a state variable of model nodes within the optimal simulations, which, as a “final common pathway,” reflects the collective outcome of the various model parameters on the E-I ratio within each node. At a neuronal level, the E-I ratio is commonly defined as the ratio, or the balance, between excitatory and inhibitory currents, potentials, and conductance onto excitatory neurons (*81*, *82*). Accordingly, in our study, we quantified the E-I ratio based on time-averaged input current onto the excitatory neurons, $\left\langle \;I_{i}^{E}\;\right\rangle$ , which reflects the net difference of the excitatory and inhibitory currents onto these neurons. Of note, our measure differs from another BNM-based measure of the E-I ratio based on model state variables, which was used in a similar previous study (*56*): the ratio of the time-average excitatory synaptic gating variable, $\left\langle \;S_{i}^{E}\;\right\rangle$ , to the time-average inhibitory synaptic gating variable, $\left\langle \;S_{i}^{I}\;\right\rangle$ . By inspecting the time series of model state variables in an example optimal simulation, we found that $I_{i}^{E}(t)$ and $S_{i}^{E}(t)/S_{i}^{I}(t)$ are positively correlated (*R*^2^ = 0.469). However, in subsequent analyses, we found notable differences between these two alternative BNM-based measures of the E-I ratio: (i) Assuming that the firing rate of excitatory neurons, $r^{E}$ , is an outcome of the E-I ratio (*61*), which indicates low versus high states of activity (*81*), we expect a measure of the E-I ratio to positively correlate with it. Accordingly, in an example simulation and using an exponential generalized linear mixed-effects model, we found that $r_{i}^{E}(t)$ correlates positively across nodes and time with both $I_{i}^{E}(t)$ (*R*^2^ = 0.977) and $S_{i}^{E}(t)/S_{i}^{I}(t)$ (*R*^2^ = 0.573) but is more strongly correlated with $I_{i}^{E}(t)$ (fig. S19). This was expected given that model Eq. 3 directly relates $I_{i}^{E}(t)$ to $r_{i}^{E}(t)$ . (ii) Next, given the optimal simulation of 40 randomly selected subjects of the PNC dataset, we performed perturbed simulations in which one of the model parameters was increased or decreased by 10%, pushing the simulation to an expected state of increased/decreased excitation/inhibition (e.g., the 10% increase in G is expected to push the system toward higher excitation). We then used paired *t* tests to compare each measure of the E-I ratio before and after the perturbation (fig. S20) and found larger effects of perturbations on $\left\langle \;I_{i}^{E}\;\right\rangle$ (mean ∣T∣ = 21.480 ± 5.383) compared to $\left\langle S_{i}^{E}\right\rangle \;/\;\left\langle S_{i}^{I}\right\rangle$ (mean ∣T∣ = 6.994 ± 2.726). This shows that $\left\langle \;I_{i}^{E}\;\right\rangle$ may be more sensitive to capture the variations in the E-I ratio caused by parameter perturbations. (iii) Last, we studied the effects of age on $\left\langle S_{i}^{E}\right\rangle \;/\;\left\langle S_{i}^{I}\right\rangle$ in a random subsample of 200 subjects from the PNC dataset (same as data used in the “Sensitivity analyses” section) and, across different model configurations, found significant increases primarily in unimodal areas, while most of the association areas showed no changes or small decreases (fig. S21). Notably, the effect of age on $\left\langle S_{i}^{E}\right\rangle \;/\;\left\langle S_{i}^{I}\right\rangle$ , compared to its effect on $\left\langle \;I_{i}^{E}\;\right\rangle$ , was less sensitive to the choice of SC, parcellation, inclusion of interhemispheric connections, and random seeds in the simulation (mean ICC: 0.999) and optimization (mean ICC: 0.963) but more sensitive to the choice of maps.

These findings, combined with the commonly used definition of the E-I ratio in experimental and theoretical research at the neuronal level, suggest that $\left\langle \;I_{i}^{E}\;\right\rangle$ may be a more direct and interpretable measure of the E-I ratio using the BNM approach compared to the alternatives used in the literature. They also highlight how modeling choices and parameters can affect the outcomes of E-I ratio changes with age.

## DISCUSSION

In this study, we used large-scale simulations of biologically realistic and individualized BNMs to estimate regional E-I ratio based on in vivo imaging data and evaluated its maturation during adolescence. We found a developmental decrease in the E-I ratio (higher inhibition or lower excitation) in the association areas, while the sensorimotor areas showed a lack of significant changes or a developmental increase. This finding was supported by imaging data from two independent datasets and through investigating both cross-sectional, interindividual age-related variations of the E-I ratio, as well as its longitudinal, within-individual changes through adolescence. Our observed pattern of regional variability in the E-I ratio development aligned with the sensorimotor-association axis of cortical organization and highlighted the divergence of early versus late developmental timing of the sensorimotor and association areas. We extensively tested the sensitivity of our findings to various modeling nuisances and choices and found that despite certain variations, the E-I ratio maturation pattern was largely robust to them. Last, we contrasted our simulation-based measure of the E-I ratio to the alternative measures used in the literature and highlighted important considerations on their interpretations.

We found a robust and replicable developmental pattern of decreased E-I ratio in the association areas, indicating a relative increase in inhibition or decrease in excitation. This observation is in line with several findings from previous animal and human studies. At the molecular level, transcriptomics and proteomics analyses have revealed periadolescence changes in the expression of genes related to excitation and inhibition, such as the NMDA receptor subunits (*83*); calcium-binding proteins parvalbumin, calretinin, and calbindin, which are expressed in different types of interneurons (*9*, *26*, *27*, *32*); and GABA_A_ receptor subunits (*28*–*31*). These molecular shifts mirror changes in neuronal functional properties. For instance, within the prefrontal cortex, there is an increase in the subunit composition of the GABA_A_ receptors, from α_2_- to α_1_-containing receptors, which have a faster decay time, resulting in faster synaptic inhibition (*31*, *32*, *84*). Consistent with this, recording of pyramidal neurons of the prefrontal cortex in nonhuman primates has indicated an increase in the strength as well as shortening of the inhibitory postsynaptic currents (*9*, *30*). At the same time, microscopic investigation of the pyramidal neurons in the prefrontal cortex across different species has revealed a marked reduction of the excitatory synaptic density during adolescence (*19*–*23*, *25*, *85*). In humans, a recent study on the PNC dataset estimated the E-I ratio in vivo by modeling multivariate patterns of FC and assessing their (dis)similarity to the FC of adults receiving alprazolam, a GABAergic agonist, and reported a significant developmental decrease in the E-I ratio, which was specific to association areas (*33*). Furthermore, magnetic resonance spectroscopy has been used in several human studies to assess the maturation of in vivo levels of glutamate and GABA, primarily in the frontal areas, yet has reported inconsistent findings (*38*–*40*, *86*–*89*). Notably, this inconsistency has been attributed to the sensitivity of lower-field scanners to macromolecule contaminants, which undermines the reliability of the findings in earlier studies (*39*). However, a recent study used 7-T scanners and found an age-related decrease in glutamate despite stable levels of GABA in the dorsolateral prefrontal cortex and decreased levels of both glutamate and GABA, despite no changes in their ratio, in the anterior cingulate and anterior insular cortices (*39*).

In contrast to the widespread decrease in the E-I ratio we observed in association regions during adolescence, in sensorimotor areas, we found an increase or no significant age-related changes of the E-I ratio. Human cortical maturation is suggested to unfold across a sensorimotor-association axis, with a differential temporal patterning indicating earlier maturation of the sensorimotor areas in contrast to later and more protracted maturation of the association cortices (*18*). In line with this, we found that the spatial pattern of E-I ratio maturation across cortical areas coaligns with the proposed sensorimotor-association axis of neurodevelopment (*18*). We additionally indicated that the genes preferentially expressed in association areas (showing a maturational decrease in the E-I ratio) are more prominent in later stages of development. The sensorimotor-association neurodevelopmental variation has been observed in cortical maturation of macrostructural features (*90*), intracortical myelination (*91*, *92*), white matter connectivity (*93*), and functional organization (*18*, *94*, *95*), parallel to the maturation of excitation and inhibition (*18*). Consistent with the findings in the prefrontal cortex (*21*, *22*), accelerated pruning of excitatory synapses around puberty has been observed in sensorimotor areas as well (*85*, *96*, *97*), although synaptic pruning in association regions is protracted and peaks later than in sensorimotor areas (*18*, *24*). In addition, the maturation of parvalbumin inhibitory interneurons in association areas is suggested to be more prolonged (*18*, *98*). Given the differences in the neurodevelopmental timing along the sensorimotor-association axis, it may be that the E-I ratio matures earlier in the sensorimotor areas before adolescence, and hence, we did not find a maturational increase in relative inhibition in these areas during our study age period. In line with our observation, two other studies using human in vivo markers of E-I ratio reported a significant increase in inhibition markers in association areas despite no significant changes in sensorimotor regions (*33*, *40*). Future research can further investigate the regional differences in the timing of E-I ratio maturation by extending our approach to a wider age range, including developmental stages before and after adolescence. The presumed hierarchical progression of E-I maturation from sensorimotor areas in early life to association areas in later stages of development is thought to have important functional consequences (*99*, *100*). Specifically, the maturation of the inhibitory circuitry in an area is mirrored by a critical period of enhanced experience-dependent plasticity, which is thought to support a shift of activity from spontaneous to stimulus evoked and in turn enhance the signal-to-noise ratio in performing task-dependent computations of that region. The hierarchical progression of this maturational cascade from sensorimotor areas in early life to association areas in later stages of development is in turn suggested to support the maturation of lower-order sensory and motor functions toward higher-order social and executive functions (*99*, *100*). Myelination, which is thought to increase near the end of these critical periods of plasticity (*99*), follows a similar hierarchical maturational trajectory (*18*), and it will be intriguing for future studies to investigate the link between the maturation of E-I ratio and myelination.

There are important methodological considerations in using BNMs to estimate E-I ratio based on imaging data. Our study extends upon a recent modeling study, which found a widespread relative increase in inhibition across the cortex, most prominently in the sensorimotor areas, by using BNMs constructed for 29 age groups of the PNC dataset and based on a template SC of an adult sample (*56*). In contrast, here, we used large-scale simulations to construct individualized BNMs (*44*, *54*, *55*), which allowed more specific simulation-based mapping of the E-I ratio in each individual subject, and this also enabled studying changes of the E-I ratio longitudinally within the same individual. In addition, individualized BNMs are shown to enhance the reliability of model parameters and fingerprinting accuracy of the simulated data (*55*). While our findings within the association areas were in agreement with the study by Zhang *et al.* (*56*), indicating a maturational decrease in the E-I ratio, they diverged in the sensorimotor areas. We suspect that this divergence can be attributed to several differences of the two studies, which, in addition to the usage of group-level versus individualized BNMs, include different simulation-based markers of the E-I ratio as well as the methodological details of image processing, modeling, and optimization. However, we presented findings that highlighted that $I_{i}^{E}$ , compared to $S_{i}^{E}/S_{i}^{I}$ , as well as model parameters, may be a more direct BNM-based marker of the E-I ratio and be closer to its common definition in the literature as the ratio, or the balance, between excitatory and inhibitory currents, potentials, and conductance onto excitatory neurons (*81*, *82*).

To further investigate the role of methodological choices and confounds in our findings, we performed extensive sensitivity analyses and showed that our key findings remain robust to such nuisances. By meta-analytically pooling the age effects on E-I ratio across different models, we found a developmental pattern consistent with our main findings. However, while the overall maturational patterns were stable, we observed notable variations in the simulation outcomes across these alternative models, highlighting the importance of modeling and analytical decisions when using BNMs to infer hidden features such as the E-I ratio, in particular at the level of individual data. One critical methodological consideration is the selection of heterogeneity maps (or their absence), which can influence the spatial pattern of the E-I ratio maturation, although in nontrivial ways. While map-free models might eliminate this potential confound, they may not be biologically valid or technologically feasible options, and we showed that they lead to worse fitting of the model to the empirical data. Specifically, a “homogeneous” map-free model assumes that the local microcircuits are uniform across the cortex, contradicting well-established regional differences, for example, in the distribution of excitatory and inhibitory neuronal subtypes (*65*, *101*). Conversely, a “node-based heterogeneous” model is too complex and difficult to fit, requiring considerably more simulations per subject. This makes it infeasible to use such a complex model within our individualized modeling approach applied to many subjects and sessions. Given challenges of map-free models, map-based heterogeneity offers a pragmatic intermediate solution that enables constrained heterogeneity of the parameters while maintaining its feasibility. Another key methodological consideration is the characteristics of the Gaussian noise incorporated in BNMs to account for the inherent stochasticity of the brain (*102*, *103*). We found that variations in the random sequence of noise influenced simulation outcomes, yet the observed effects of age on the E-I ratio remained largely robust. This highlights that future BNM studies should explicitly take the often-overlooked impacts of noise random sequence into account, for example, by repeating the simulations across multiple random seeds to assess the stability of their findings.

Recognizing the influence of modeling choices on simulation outcomes, we emphasize the need for future research to systematically explore and characterize these effects. While dedicated methodological studies should continue investigating how different modeling decisions shape BNM-derived measures (*57*, *58*), studies applying BNMs to specific neurobiological questions should also assess the impact of alternative modeling choices on their reported findings. However, comprehensive sensitivity analyses can be computationally demanding, making it impractical to explore all sources of variability across large datasets. To address this, future research should consider using more efficient simulation implementations, such as the GPU-based approach introduced in this study, or developing methods that estimate modeling influences without running numerical simulations, such as analytical approximations (*66*) or deep learning–based methods (*104*).

Given the methodological flexibility of BNM paradigms, establishing optimal methodological choices in mapping the E-I ratio using BNMs at the individual level remains an open challenge. Therefore, it will be crucial for the future research to investigate the following: (i) Comprehensively assessing how modeling choices may affect the simulation-derived E-I ratio measures. (ii) Developing approaches to enable more complex models while maintaining their feasibility and avoiding overfitting. Such models may include a “node-based heterogeneous” model or models that are more biologically detailed via incorporation of layer-wise neuronal subtypes and feedback/feedforward connections (*105*, *106*), the modulatory neurotransmitter systems (*46*), or the dynamics of α-amino-3-hydroxy-5-methyl-4-isoxazolepropionic acid receptors (*107*). (iii) Evaluating the replicability of our findings in alternative datasets, particularly with higher imaging quality and more extensive follow-ups, as well as testing the fitting of BNMs to additional empirical measures such as magneto/electroencephalography (*44*, *108*). (iv) Experimentally validating the E-I ratio measures obtained through fitting of BNMs to the fMRI data against empirical measures obtained using electrical recordings or evaluating them in response to excitatory/inhibitory interventions.

Overall, this study provides in vivo evidence, based on individualized BNMs, of a replicable and robust decrease in cortical E-I ratio in association areas during adolescence. The normative maturation of E-I ratio is suggested to have important functional consequences (*18*, *99*) and its dysmaturation is believed to be associated with various neurodevelopmental disorders (*11*, *12*). For example, the neurodevelopmental model of schizophrenia suggests that aberrant cortical maturation, particularly in the development of excitatory and inhibitory functions, may contribute to the emergence of the disease later in life (*12*, *13*, *17*, *109*). Future studies should investigate the clinical relevance of adolescent maturation of E-I ratio in relation to the risk and diagnosis of neurodevelopmental disorders such as schizophrenia, as this could potentially offer biomarkers for early detection and intervention.

## MATERIALS AND METHODS

This research complies with the ethical regulations as set by The Independent Research Ethics Committee at the Medical Faculty of the Heinrich Heine University Düsseldorf (study number 2018-317). We used previously published data from sources that have received ethics approval from their respective institutions (*52*, *53*, *110*–*112*).

### Participants

We studied adolescents from two population-based datasets, including the cross-sectional PNC (*52*, *110*, *111*) and the longitudinal IMAGEN dataset (*53*). We selected subjects and follow-up sessions within the age range of 10 to 19 years. In the IMAGEN cohort, within this age range, phenotypic assessments were conducted at the ages of 14, 16, and 19 and imaging data were acquired at the ages of 14 and 19. Next, we excluded subjects with poor quality of raw or processed imaging data, as detailed below in the “Image processing and quality control” section. In the IMAGEN, the subjects were excluded if the imaging data had poor quality in any of the baseline or follow-up sessions, except for the DWI data for which subjects were selected on the basis of the image quality of the follow-up session. Our final sample consisted of 752 adolescents from the PNC (409 female; mean age: 15.3 ± 2.4 years) and 149 participants from IMAGEN (72 female; baseline mean age: 14.4 ± 0.4 years; follow-up mean age: 18.9 ± 0.5 years). The PNC data were collected at a single center in Philadelphia, while the IMAGEN data were acquired in five different centers across Europe, in Dresden (*n* = 58), Paris (*n* = 45), Mannheim (*n* = 30), London (*n* = 14), and Dublin (*n* = 2). The PNC included subjects with European American (*n* = 351), African American (*n* = 332) and mixed/other (*n* = 69) ethnicities, and the IMAGEN cohort consisted of subjects with Caucasian (*n* = 134), mixed (*n* = 12), and non-Caucasian (*n* = 3) ethnicities.

### Image acquisition

T1w, rs-fMRI, and DWI data were acquired using 3-T scanners from different manufacturers (PNC: Siemens; IMAGEN: Siemens, Philips, General Electric). For more details on the image acquisition parameters, we refer the reader to the respective publications for each dataset [PNC: table 1 in (*52*); IMAGEN: table S5 in (*53*)]. Of particular relevance to our study, the repetition and acquisition times of the rs-fMRI images were respectively 3 s and 6:18 min in the PNC and 2.2 s and 6:58 min in the IMAGEN.

### Image processing and quality control

#### T1w structural magnetic resonance imaging

T1w MRI images were processed using the recon-all command of FreeSurfer (version 7.1.1; https://surfer.nmr.mgh.harvard.edu/), which includes brain extraction, registration to standard space, tissue segmentation, and cortical surface reconstruction (*113*, *114*). The quality of the FreeSurfer output was controlled on the basis of the Euler characteristic, which represents the number of cortical surface defects before correction. We excluded outlier subjects with Euler characteristic greater than $Q_{3}+1.5\times \text{IQR}$ (interquartile range) of their cohort (*115*). The FreeSurfer output and raw T1w images were subsequently used in the pipelines of rs-fMRI and DWI processing.

#### Resting-state functional magnetic resonance imaging

rs-fMRI images were preprocessed using fMRIprep (version 22.0.0; https://fmriprep.org/en/stable/), which performed brain extraction, image registration and motion correction, estimation of confounds, and when possible, susceptibility distortion and slice time corrections (*116*). The latter two steps were omitted in the IMAGEN data given the unavailability of slice timing and variability of field-map formats across centers. We next further processed the output of fMRIprep, which involved the following steps: (i) applying Schaefer-100 parcellation (*60*) by taking the average signal of all vertices within each parcel at each time point, (ii) removing the first three volumes, (iii) high-pass temporal filtering >0.013 Hz, (iv) regressing out confounds including the average signal of white matter and cerebrospinal fluid voxels as well as 24 motion parameters (translation and rotation in the three directions, in addition to their squares, derivatives, and squares of derivatives) (*117*), and (v) scrubbing motion outliers, defined on the basis of root mean squared translation >0.25 mm. The scrubbing was done by setting the signal in motion outlier volumes to zero while *Z*-scoring the rest of the volumes. This approach, compared to discarding the motion outliers, preserves the temporal structure of the BOLD signal, which is important in calculating dynamic FC.

We excluded subjects/sessions with high rs-fMRI in-scanner motion defined as less than 4 min of scan remaining after scrubbing motion outlier volumes or a time-averaged root mean square >0.2 mm. In addition, we performed visual quality control of the fMRIprep output and excluded subjects with gross misregistration or incomplete field of view.

##### Functional connectivity

FC was calculated as the Pearson correlation of BOLD signal time series between the cortical areas.

##### Functional connectivity dynamics

The FCD matrix represents the temporal variability of the dynamic patterns of FC computed across sliding windows of time (*118*). To compute the FCD matrix, we initially calculated time-resolved FC matrices of sliding windows (PNC: size of 30 s and step of 6 s; IMAGEN: size of 30.8 s and step of 4.4 s). We discarded edge windows and windows with ≥50% motion outliers. Subsequently, we computed FCD as the correlation between lower triangular parts of window FC patterns. The distribution of values within the FCD matrix represents the amount of recurrence of time-resolved FC patterns.

#### Diffusion-weighted imaging

DWI images were processed using Micapipe (version 0.1.1; https://micapipe.readthedocs.io/en/latest/) (*119*), which combines tools from FSL (version 6.0.0) (*120*) and MRTrix3 (version 3.0.0) (*121*). This pipeline performed DWI processing steps including rigid-body alignment of images, Marchenko-Pastur principal component analysis denoising, Gibbs ringing correction, motion and eddy current-induced distortion correction, nonuniformity bias correction, registration to the processed structural image, brain mask generation, and estimation of fiber orientation distributions using spherical deconvolution. Then, on each image, probabilistic tractography was performed using the iFOD2 algorithm to estimate 10 million streamlines (*122*). In addition, a track density image was computed using the iFOD1 algorithm with 1 million streamlines, which was used for the quality control (*123*). The quality control of the tractograms was done by visual inspection of the tractogram density images.

#### Structural connectivity

The SC matrix for each subject was created using Micapipe by parcellating the tractogram using the Schaefer-100 parcellation map (*60*) non-linearly registered to the DWI space. We subsequently normalized each SC matrix by division by its mean × 100, resulting in an equal mean of 0.01 in all SCs.

In addition to subject- and session-specific SCs of the adolescents, we used higher-quality DWI data (3 T, three shells, 140 directions) of an adult sample of 50 healthy volunteers (MICs dataset, 23 female; mean age: 29.5 ± 5.6 years) to construct a template SC (*112*). To do so, we obtained the SC of individual MICs subjects preprocessed using Micapipe and calculated a group-averaged SC by taking the median of streamline counts in each edge. The template SC was subsequently normalized by its mean × 100, similar to the subject- and session-specific SCs of the adolescents.

In the individualized models reported in the main analyses, we used subject-specific SCs, but the adult template SC was used in the sensitivity analysis on the confounding effects of interindividual variability in SC. Of note, in IMAGEN, given the lower quality of tractograms in the baseline session, the main analyses were performed by using the follow-up SC of each subject for the modeling of functional data in both the baseline and follow-up sessions. However, in a supplementary analysis, we additionally performed simulations using session-specific SCs, within a subset of the IMAGEN subjects with adequate quality of tractograms in both sessions (*n* = 110, 52 female).

### Biophysical network modeling

Next, we performed BNM simulation-optimization at the level of each individual subject/session (Fig. 1).

#### Model simulation

We simulated the spontaneous neuronal activity of 100 cortical regions from the Schaefer atlas *(**60*) as network nodes regulated by the reduced Wong-Wang model and interconnected through the SC (*61*). In short, this model describes the activity of large ensembles of interconnected excitatory and inhibitory spiking neurons in each area by a dynamic mean field model as a reduced set of dynamic equations governing the activity of coupled excitatory and inhibitory pools of neurons. In this reduced model, the excitatory synaptic currents are mediated by the NMDA receptors and the inhibitory synaptic currents are mediated by the GABA_A_ receptors. Within each cortical region, the excitatory and inhibitory neuronal pools are interconnected, and between regions, the excitatory neuronal pools are coupled through a scaled SC matrix.

##### Model equations

The model is mathematically described by a set of dynamic equations (*61*). The total input current (in nA) to each excitatory and inhibitory neuronal pool of each cortical node *i*, $I_{i}^{\left(E/I\right)}$ , is calculated as

$$I_{i}^{E}\left(t\right)=W^{E}I_{b}+w_{i}^{\text{EE}}S_{i}^{E}\left(t\right)+GJ_{\text{NMDA}}\sum_{j=1}^{N}C_{\mathrm{ij}}S_{j}^{E}\left(t\right)-w_{i}^{\text{IE}}S_{i}^{I}\left(t\right)\;$$ (1)

$$I_{i}^{I}\left(t\right)=W^{I}I_{b}+w_{i}^{\text{EI}}S_{i}^{E}\left(t\right)-w^{\text{II}}S_{i}^{I}\left(t\right)$$ (2)

where $W^{E}I_{b}$ = 0.382 nA and $W^{I}I_{b}$ = 0.267 nA are the baseline input currents; $S_{i}^{\left(E/I\right)}$ denote the synaptic gating variables; *n* = 100 is the number of nodes; C is the SC matrix, which together with the NMDA receptor conductance, $J_{\text{NMDA}}$ = 0.15 nA, and G (global coupling), a free parameter of the model, determines the excitatory input current transmitted from the other nodes; $w_{i}^{\text{EE}}$ is the recurrent excitatory connection weight; $w_{i}^{\text{EI}}$ indicates the excitatory-to-inhibitory connection weight; $w_{i}^{\text{IE}}$ is the inhibitory-to-excitatory connection weight; and $w^{\text{II}}$ = 1.0 denotes recurrent inhibitory connection weight. The local connection weights $w_{i}^{\text{EE}}$ , $w_{i}^{\text{EI}}$ , and $w_{i}^{\text{IE}}$ can vary across nodes and simulations through the free parameters of the model and the FIC, as described below.

The total input current received by each neuronal pool is subsequently transferred to $r_{i}^{\left(E/I\right)}$ , firing rates in Hz, using the sigmoidal neural response function, $H^{\left(E/I\right)}$

$$r_{i}^{E}\left(t\right)=H^{E}\left(I_{i}^{E}\right)=\frac{a^{E}I_{i}^{E}\left(t\right)-b^{E}}{1-e^{-d^{E}\left[a^{E}I_{i}^{E}\left(t\right)-b^{E}\right]}}$$ (3)

$$r_{i}^{I}\left(t\right)=H^{I}\left(I_{i}^{I}\right)=\frac{a^{I}I_{i}^{I}\left(t\right)-b^{I}}{1-e^{-d^{I}\left[a^{I}I_{i}^{I}\left(t\right)-b^{I}\right]}}$$ (4)

where $a^{E}$ = 310 nC^−1^ and $a^{I}$ = 615 nC^−1^ determine the slope of $H^{\left(E/I\right)}$ ; $b^{E}$ = 0.403 nA and $b^{I}$ = 0.288 nA define the thresholds above which the firing rates increase linearly with the input currents; and $d^{E}$ = 0.16 and $d^{I}$ = 0.087 determine the shape of $H^{\left(E/I\right)}$ curvature around $b^{\left(E/I\right)}$.

Last, the synaptic gating variables, $S_{i}^{\left(E/I\right)}$ , follow

$$\frac{dS_{i}^{E}\left(t\right)}{\mathrm{dt}}=-\frac{S_{i}^{E}\left(t\right)}{\tau_{E}}+\left[1-S_{i}^{E}\left(t\right)\right]\gamma r_{i}^{E}\left(t\right)+\sigma \nu_{i}\left(t\right)$$ (5)

$$\frac{dS_{i}^{I}\left(t\right)}{\mathrm{dt}}=-\frac{S_{i}^{I}\left(t\right)}{\tau_{I}}+r_{i}^{I}\left(t\right)+\sigma \nu_{i}\left(t\right)$$ (6)

where $S_{i}^{E}$ is mediated by NMDA receptors with a decay time constant $\tau_{E}$ = 0.1 s and γ = 0.641, and $S_{i}^{I}$ is mediated by GABA receptors with a decay time constant $\tau_{I}$ = 0.01 s. $\nu_{i}\left(t\right)$ is uncorrelated standard Gaussian noise with an amplitude of σ = 0.01 nA. $S_{i}^{\left(E/I\right)}$ is subsequently bound within the range [0, 1]. Note that the fixed parameters in Eqs. 1 to 6 are based on a previous paper by Deco *et al.* (*61*).

##### Model free parameters

The model is controlled by 15 free parameters, including G , as well as bias terms and coefficients for a fixed set of six biological maps, which together determine the regional values of $w_{i}^{\text{EE}}$ and $w_{i}^{\text{EI}}$ . More specifically, $w_{i}^{p},p\in \left\{\;\text{EE},\text{EI}\;\right\}$ is calculated as

$$w_{i}^{p}=w_{b}^{p}\cdot \left(1+\sum_{k=1}^{6}c_{k}^{p}M_{\mathrm{ki}}\right)$$ (7)

where $w_{b}^{p}$ , the bias term, and $c^{p}$ , a vector of six coefficients, are free parameters. M is a 6 by 100 matrix including the *Z*-scored biological maps in Schaefer-100 parcellation (fig. S1). These maps were based on independent samples of healthy individuals, obtained from neuromaps (*74*) and Hansen *et al.* (*70*) and included the following: (i) group-averaged T1w/T2w ratio map of the Human Connectome Project dataset (*68*, *69*, *124*), (ii) group-averaged cortical thickness map of the Human Connectome Project dataset, (iii) FC G1 (*72*), (iv) principal axis of Allen Human Brain Atlas gene expression data (Gene PC1) (*71*, *73*), (v) NMDA receptor density positron emission tomography map (*67*, *70*), and (vi) GABA_A/BZ_ receptor density positron emission tomography map (*70*, *75*). The resulting $w_{i}^{\left(\text{EE}/\text{EI}\right)}$ maps were subsequently shifted if needed to ensure $\text{min}\left[w_{i}^{\left(\text{EE}/\text{EI}\right)}\right]$ ≥ 0.001. We used the following ranges for the model free parameters: G = [0.5, 4.0] and $w_{b}^{\left(\text{EE}/\text{EI}\right)}$ = [0.05, 0.75]. The range of coefficients for each map was defined as $\left[\frac{-1}{\text{max}\left(\text{map}\right)},\frac{-1}{\text{min}\left(\text{map}\right)}\right]$ , corresponding to *c*_T1w/T2w_ = [−0.48, 0.59], *c*_cortical thickness_ = [−0.40, 0.39], *c*_FC G1_ = [−0.59, 0.72], *c*_Gene PC1_ = [−0.36, 0.48], *c*_NMDA_ = [−0.49, 0.42], and *c*_GABAa/bz_ = [−0.30, 0.32].

##### Feedback inhibition control

FIC was used to determine the regional values of $w_{i}^{\text{IE}}$ in each simulation, given the SC and other model parameters. The FIC algorithm aims to maintain a state of E-I balance in each region by adjusting $w_{i}^{\text{IE}}$ to satisfy an excitatory firing rate close to 3 Hz, which is suggested to be within the biological range (*61*). We used a two-stage implementation of the FIC by combining the original numerical implementation (*61*) with an analytical solution proposed by Demirtaş *et al.* (*66*). The latter solution analytically solves for $w_{i}^{\text{IE}}$ to satisfy the self-consistency of the model equations under the steady-state condition with $\left\langle \;r_{i}^{E}\;\right\rangle$ ≈ 3 Hz, corresponding to $\left\langle \;S^{E}\;\right\rangle$ ≈ 0.164757 and $\left\langle I^{E}\right\rangle$ ≈ 0.37738 nA

$$w_{i}^{\text{IE}}=\frac{W^{E}I_{b}+w_{i}^{\text{EE}}\left\langle S^{E}\right\rangle +{\mathrm{GJ}}_{\mathrm{NMDA}}\left\langle S^{E}\right\rangle -\left\langle I^{E}\right\rangle }{\left\langle S_{i}^{I}\right\rangle }$$ (8)

in which the steady-state inhibitory synaptic gating variable $\left\langle S_{i}^{I}\right\rangle =H^{I}\left(\langle I_{i}^{I}\rangle \right)\tau_{I}$ was estimated by solving for $\left\langle \;I_{i}^{I}\;\right\rangle$ in

$$W^{I}I_{b}+w_{i}^{\text{EI}}\left\langle S^{E}\right\rangle -H^{I}\left(\langle I_{i}^{I}\rangle \right)\tau_{I}-\left\langle I_{i}^{I}\right\rangle =0$$ (9)

Subsequently, analytical estimates of $w_{i}^{\text{IE}}$ values were fed into the numerical implementation of FIC and were adjusted numerically (*61*). In this approach, given the analytical estimates of $w_{i}^{\text{IE}}$ , the model Eqs. 1 to 6 are numerically integrated for a short period of 10 s and, subsequently, the average input current to the excitatory pool of each brain region, $\left\langle \;I_{i}^{E}\;\right\rangle$ , is calculated. If $\left\langle \;I_{i}^{E}\;\right\rangle -\frac{b_{E}}{a_{E}}$ in a region exceeded the target −0.026 by more than 0.005 nA, $w_{i}^{\text{IE}}$ is up/down-regulated when the input current is higher/lower than the target, and the simulation is repeated with the adjusted $w_{i}^{\text{IE}}$ values in the next trial. This procedure is repeated for 10 trials or until the FIC target is satisfied in all nodes. Note that the maximum number of FIC numerical adjustment trials used here is lower than that of the original implementation to facilitate the scaling of the simulations. Furthermore, as the initial $w_{i}^{\text{IE}}$ values are estimated analytically rather than being fixed to 1 (as was done in the original implementation), a smaller number of trials will be needed.

##### Hemodynamics model

The simulated synaptic activity of the excitatory population of each node, $S_{i}^{E}$ , was subsequently fed to the Balloon-Windkessel model of hemodynamics to simulate the BOLD signal (*125*). This model is mathematically described by the following system of differential equations with state variables x (vasodilatory signal), f (blood inflow), v (blood volume), and q (deoxyhemoglobin content)

$$\frac{\mathrm{dx}\left(t\right)}{\mathrm{dt}}=S^{E}\left(t\right)-\kappa x\left(t\right)-\gamma \left[f\left(t\right)-1\right]$$ (10)

$$\frac{\mathrm{df}\left(t\right)}{\mathrm{dt}}=x\left(t\right)$$ (11)

$$\tau \frac{\mathrm{dv}\left(t\right)}{\mathrm{dt}}=f\left(t\right)-v^{\frac{1}{\alpha }}\left(t\right)$$ (12)

$$\begin{array}{c} \tau \frac{\mathrm{dq}\left(t\right)}{\mathrm{dt}}=\frac{f\left(t\right)}{\rho }\left\{\;1-\left[\;{\left(1-\rho \right)}^{\frac{1}{f\left(t\right)}}\right],\;\right\}-\frac{q\left(t\right)v^{\frac{1}{\alpha }}\left(t\right)}{v\left(t\right)} \end{array}$$ (13)

where κ = $\frac{1}{0.65}$ s^−1^ is the rate of signal decay, γ = $\frac{1}{0.41}$ s^−1^ is the rate of flow-dependent elimination, τ = 0.98 s is the hemodynamic transmit time, α = 0.32 is Grubb’s exponent, and ρ = 0.34 is the resting oxygen extraction fraction. These parameters were based on a previous paper by Friston *et al.* (*125*). Last, on the basis of the model state variables, the BOLD signal is calculated as

$$\begin{array}{c} B\left(t\right)=V_{0}\left\{k_{1}\left[1-q\left(t\right)\right]+k_{2}\left[1-\frac{q\left(t\right)}{v\left(t\right)}\right]+k_{3}\left[1-v\left(t\right)\right]\right\} \end{array}$$ (14)

in which $V_{0}$ = 2% is the resting blood volume fraction (*125*), and $k_{1}$ = 3.72, $k_{2}$ = 0.527, and $k_{3}$ = 0.53 are dimensionless parameters that were derived for 3-T scans (*66*, *126*). Last, the simulated BOLD signal was downsampled to match the repetition time of the empirical rs-fMRI data, i.e., 3 s for the PNC and 2.2 s for IMAGEN.

##### Numerical integration of the models

For each simulation, the model equations were numerically integrated using the Euler method with a time step of 0.1 ms for the neuronal model (Eqs. 1 to 6) and a time step of 1 ms for the hemodynamic model (Eqs. 10 to 13). The model simulations were performed using in-house code (https://github.com/amnsbr/bnm_cuda and https://cubnm.readthedocs.io; see the “Data and materials availability” section for more details) on GPUs at JURECA-DC (*127*), Raven, or Juseless high performance/throughput computing systems. This GPU implementation enabled efficient parallelization of calculations for individual simulations (across GPU “blocks”) and the regions within each simulation (across GPU “threads”). To match the duration of empirical rs-fMRI scans, the simulations were done for a biological duration of 450 s, from which the first 30 s was discarded to ensure that the BNM system’s state has stabilized.

#### Model evaluation

The goodness of fit of the simulated BOLD signal given a set of candidate parameters and SC matrix to a target empirical BOLD signal was evaluated on the basis of three measures of static and dynamic FC, following previous studies (*56*, *65*):

##### Static edge-level FC

The simulated FC was calculated as the correlation of simulated BOLD signal time series between nodes. The correspondence of simulated and empirical FC patterns was evaluated by calculating the Pearson correlation coefficient between the lower triangles of the matrices (FC_corr_), with larger values representing higher correspondence.

##### Static global FC

The absolute difference of the averaged simulated and empirical FC matrices across all the lower triangular edges (FC_diff_) was calculated to assess the similarity of global FC strength, with smaller values showing higher correspondence.

##### Dynamic FC

The simulated FCD matrix was constructed by calculating the correlation of FC patterns between sliding windows of simulated BOLD signals, as described previously for the empirical data. The correspondence of simulated and empirical FCD distributions was calculated as the Kolmogorov-Smirnov (KS) distance of their lower triangular parts (FCD_KS_), with smaller values showing higher similarity of the distributions.

Subsequently, these measures were combined into a single measure of goodness of fit as FC_corr_ – FC_diff_ − FCD_KS_. Of note, in goodness-of-fit calculations, following Demirtaş *et al.* (*66*), we excluded the interhemispheric connections. However, we also performed a robustness analysis in which these connections were included in the goodness-of-fit calculations.

#### Parameter optimization

The model’s free parameters (*n* = 15) were fit to the empirical data of each subject/session using the CMA-ES optimization algorithm (*62*–*64*). CMA-ES is an efficient evolutionary optimization algorithm that features a set of Λ particles exploring the parameter space collaboratively in an iterative process. The particles from each iteration, which are individual simulations with different free parameters, are regarded as a generation from which only the best particles are selected to form the descendant population of the next generation. Specifically, at each generation, the cost function of each particle is calculated, as described below. Then, a weighted mean of the best fitting ⌊Λ/2⌋ particles is calculated. Then, a new generation of particles is created by taking Λ samples from a multivariate normal distribution centered around the weighted mean of the best fitting ⌊Λ/2⌋ particles from the previous generation. The covariance is determined by a matrix that is updated to take the location of the current best points into account. In this way, the search distribution is adapted iteratively toward a concentration around the optimal solutions. This iterative process is continued for a maximum of 80 generations, following Wischnewski *et al.* (*64*), and eventually, the optimal point across all generations is selected as the optimal parameters for the best fit of the simulation to the given target empirical data. We also applied an early termination rule in which the iteration was stopped if there was no improvement in the cost function greater than 0.005 over the past 30 generations.

The optimization goal was to maximize the goodness of fit while minimizing a penalty term. Particles were penalized if (i) the parameter of sampled particles fell outside the prespecified ranges, in which case the parameters were corrected and a penalty was added (*63*), or (ii) the $\left\langle \;r_{i}^{E}\;\right\rangle$ was outside the range of 2 to 4 Hz in any node, indicating the insufficiency of the FIC. For the latter, the FIC penalty was calculated as

$$\text{FIC}\;\text{penalty}=\frac{2}{n}\sum_{i=1}\left(1-e^{-0.05|\left\langle r_{i}^{E}\right\rangle -3|}\right)$$ (15)

in which n is the number of nodes, and the summation is done across nodes with out-of-range $\left\langle \;r_{i}^{E}\;\right\rangle$ . Of note, we refrained from setting the success of FIC as a hard constraint, but through this penalty term, applied it as a soft constraint, to allow for interindividual variability of E-I balance while keeping it within a biologically viable range (*61*).

Given the relatively high dimensionality of the optimization problem in our model and to sufficiently cover the large parameter space, we chose Λ = 210 in the CMA-ES. Consequently, with maximum 80 generations, this involved performing a maximum of 16,800 simulations per run for each subject/session, which necessitated an efficient GPU-based implementation. In addition, for each given subject/session, the model simulation-optimization was repeated twice using different random seeds of the optimization to assess and reduce the risk of local optima. We then took the best fitting simulation across the two runs of each subject/session as their optimal simulation for the next step.

#### Estimation of the E-I ratio in silico

Thus far, we described the procedure for deriving the optimal parameters that result in a simulation best fitting to the empirical rs-fMRI data of a given subject/session using individualized BNMs. Then, given the optimal simulation for each subject/session, we extracted an in silico measure of regional E-I ratio. To do so, we calculated the average of total input current to the excitatory neurons of each region after discarding the initial 30 s of the simulation. This measure can be interpreted as an in silico marker of the regional E-I ratio, as $I_{i}^{E}$ (Eq. 1) results from the combination of excitatory input currents to each node (from itself and from the excitatory neurons of the other nodes through the SC) balanced by the inhibitory currents from the inhibitory neuron of the same node. Therefore, an increase in $\left\langle \;I_{i}^{E}\;\right\rangle$ can be interpreted as an increase in E-I ratio, i.e., a relative increase in excitation or decrease in inhibition. Furthermore, using a similar approach, we calculated $\left\langle S_{i}^{E}\right\rangle \;/\;\left\langle S_{i}^{I}\right\rangle$ as an alternative marker of the E-I ratio used in a previous study (*56*).

#### Perturbed simulations

We performed a control analysis to assess the effect of known perturbations in the model parameters on the alternative markers of the E-I ratio, namely, $\left\langle \;I_{i}^{E}\;\right\rangle$ and $\left\langle S_{i}^{E}\right\rangle \;/\;\left\langle S_{i}^{I}\right\rangle$ . In this analysis, we randomly selected 40 subjects of the PNC dataset and, for each subject, given their optimal simulations, we performed perturbed simulations in which one of the model parameters ( G , $w^{\text{EE}}$ , $w^{\text{EI}}$ , or $w^{\text{IE}}$ ) was increased or decreased by 10%, while the other three parameters were fixed to the optimal values. Notably, in these simulations, when $w^{\text{IE}}$ was not perturbed, it was not readjusted using FIC and was fixed to the $w^{\text{IE}}$ values obtained by the FIC run on the original optimal simulation. Similarly, perturbation of $w^{\text{IE}}$ was done by a 10% increase or decrease of these original $w^{\text{IE}}$ values. This was to ensure that only one parameter in the model is perturbed and, therefore, the net direction of the effect of perturbation on the E-I ratio is predictable. Last, the effect of perturbation on the E-I ratio markers in each subject was assessed using paired *t* tests comparing the marker across nodes before and after the perturbation.

### Statistical analyses

#### Age effects

Given the in silico regional measures of the E-I ratio for each subject/session, $\left\langle \;I_{i}^{E}\;\right\rangle$ , we performed group-level univariate statistical analyses to investigate the effects of age on these measures. Linear regression models were used to study the effect of age on the E-I ratio, with the goodness of fit, sex, and rs-fMRI in-scanner motion (based on time-averaged root mean squared translation) as confounds. In IMAGEN, longitudinal variation of E-I measures across the two sessions was assessed using similar linear mixed-effects regression models with random intercepts per subject and inclusion of site as an additional confound. In each model, we excluded outliers with a dependent variable ≥3 SDs above/below the mean. We adjusted for multiple comparisons across regions using FDR based on the Benjamini/Hochberg method (*q* < 0.05). Similar models were used to investigate the effects of age on optimal model parameters and $\left\langle S_{i}^{E}\right\rangle \;/\;\left\langle S_{i}^{I}\right\rangle$ . We used statsmodels (https://www.statsmodels.org/stable/index.html) (*128*) to perform regressions and FDR adjustment.

#### Within-sample stability of age effects using subsampling

In each dataset, we randomly selected 100 subsamples of the subjects (stratified by sex and age group; *n* within each subsample: 376 in PNC and 74 in IMAGEN) and investigated the effects of age on the E-I ratio separately in each subsample. Subsequently, we calculated the correlation of unthresholded age effect maps between all pairs of subsamples and reported its distribution as a measure of within-sample stability.

#### Spatial association of maps

We evaluated the spatial association of an E-I ratio maturation map *X* with a target map *Y* (another E-I ratio maturation map or a brain feature map) using Pearson correlation. In the comparison of two E-I ratio maturation maps, we additionally used cosine similarity given that Pearson correlation is insensitive to mean shifts and, thus, the changes in direction of the age effects. To account for spatial autocorrelation, the statistical significance of these associations was assessed by constructing nonparametric null distributions of Pearson correlation or cosine similarity calculated between *X* and spun surrogates of the target map *Y*. Spin permutation was implemented at the parcel level using the ENIGMA Toolbox (https://github.com/MICA-MNI/ENIGMA) (*129*) and based on 1000 permutations. This approach was used in assessing (i) the between-sample replicability of E-I ratio maturation between the PNC and IMAGEN datasets, (ii) the stability of E-I ratio maturation within a subsample of 200 subjects from the PNC dataset across the sensitivity analyses compared to the effects observed in the main run, and (iii) the spatial alignment of E-I ratio maturation maps (based on the main or alternative runs) with the maps determining the heterogeneity of regional parameters, as well as the sensorimotor-association axis of cortical organization described in a recent study by Sydnor *et al.* (*18*) and its components or their substitutes (some overlapping with the regional parameter heterogeneity maps) (fig. S6 and table S2) (*68*, *69*, *71*–*73*, *130*–*136*).

#### Distribution of the E-I ratio maturation maps across the canonical resting-state networks

We assessed the association of the E-I ratio maturation maps with the map of seven canonical resting-state networks (*76*) using one-way analysis of variance (ANOVA) with post hoc Bonferroni-corrected independent *t* tests. To control for spatial autocorrelation, we assessed the statistical significance of resulting *F* and *T* statistics using null distributions generated from 1000 spun surrogates based on parcel-level spinning implemented in the ENIGMA Toolbox (*129*).

#### Partial least squares regression of gene expressions and their developmental enrichment

Regional microarray expression data were obtained from six postmortem brains (one female; age: 24.0 to 57.0) provided by the Allen Human Brain Atlas (https://human.brain-map.org) (*71*). Data were processed with the abagen toolbox (https://abagen.readthedocs.io/en/stable/) (*73*) using the Schaefer-100 atlas (*60*). Gene expression data from the right hemisphere were excluded because of the sparsity of samples and a large number of regions with no expression data. We subsequently used scikit-learn (https://scikit-learn.org/stable/) (*137*) and performed partial least squares regression analysis to identify gene expression patterns with high spatial coalignment with the E-I ratio maturation maps within the left hemisphere. After selecting the top 500 genes with the highest absolute weights, we divided them into two sets of positively and negatively associated genes. Subsequently, using an online tool, we performed developmental specific expression analysis of these genes (http://doughertytools.wustl.edu/CSEAtool.html) (*77*). This tool uses Fisher’s exact test to assess the overlap between the set of provided genes and predefined sets of genes, which are up-regulated in each developmental stage and brain structure, identified on the basis of the BrainSpan Atlas of the Developing Human Brain (http://www.brainspan.org). Here, for each set of genes, we reported the negative log of *P* values based on the specificity index (pSI) threshold of 0.05 within the cerebral cortex and FDR adjusted across the developmental stages. To assess the statistical significance of the observed developmental enrichment pattern, we constructed null distributions of negative log values across developmental stages and positively and negatively associated genes by repeating the procedure described above for 1000 spun surrogate maps of E-I ratio maturation and performing developmental specific expression analysis on the resulting null sets of genes positively and negatively associated with the surrogate maps. The resulting spin-permutation *P* values were subsequently FDR adjusted.

#### Test-retest reliability of E-I ratio across simulations

In the sensitivity analyses, we measured the node-level test-retest reliability of E-I ratio across simulations of the same subject between two simulation runs by measuring the median absolute deviation ICC of each region between the alternative simulations and across subjects. We interpreted ICC values as poor (<0.50), moderate (0.50 to 0.75), or good (≥0.75). To assess the effect of age on ICC measures, the ICC was additionally calculated separately in the younger and older subgroups of the PNC subsample subjects (split by median age), and the resulting ICC values across nodes were compared between the two age groups using paired *t* test.

#### Association of optimal model parameters across subjects

The association of optimal regional parameters ( $w^{\text{EE}}$ , $w^{\text{EI}}$ , and $w^{\text{IE}}$ ) across subjects and nodes was tested via linear mixed-effects regressions with random intercepts and slopes per each node. These regressions were performed using the lme4 R package (*138*). In addition, we used Pearson correlation to test the association of the optimal G with the mean of optimal regional parameters.

#### Association of model state variables

We randomly selected one subject from the PNC dataset and evaluated the association of state variables within its optimal simulation across time points and nodes. The model state variables $r_{i}^{E}(t)$ , $I_{i}^{E}(t)$ , and $S_{i}^{E}(t)/S_{i}^{I}(t)$ were sampled every repetition time after the initial 30 s of the simulation was removed. Subsequently, as $S_{i}^{E}(t)/S_{i}^{I}(t)$ approaches infinity when $S_{i}^{I}(t)$ is close to zero, we excluded the data points at the top 2.5 percentile of $S_{i}^{E}(t)/S_{i}^{I}(t)$ . Subsequently, we used mixed-effects models with or without a logarithmic linking function to test the linear or exponential associations of $I_{i}^{E}(t)$ with $r_{i}^{E}(t)$ , $S_{i}^{E}(t)/S_{i}^{I}(t)$ with $r_{i}^{E}(t)$ , and $S_{i}^{E}(t)/S_{i}^{I}(t)$ with $I_{i}^{E}(t)$ and reported the results of the model with a lower Akaike information criterion. The mixed-effects models included random intercepts and slopes and were implemented using the lme4 R package (*138*) and its Python interface in the pymer4 package.

#### Random-effects meta-analysis of age effects across alternative modeling configurations

The sensitivity analyses described below resulted in several E-I ratio maturation maps based on the alternative configurations used for the BNM simulation-optimization runs. We performed random-effects meta-analyses, independently for each parcel and across seven main and alternative runs of map-based models (reported in Fig. 5, A to E), to calculate their pooled effects. The effect sizes used in these meta-analyses were partial correlations of age with the E-I ratio (after removing outliers and controlling for the goodness of fit, sex, and in-scanner rs-fMRI motion). The heterogeneity of effect sizes in each parcel was assessed using the *I*^2^ index and Cochran *Q* test, where *P*(*Q*) < 0.05 indicates significant heterogeneity. In addition to the main meta-analyses across map-based models, we performed supplementary meta-analyses in which two map-free (“homogeneous” and “node-based heterogeneous”) models were additionally included. The PyMARE (https://pymare.readthedocs.io/en/latest/) package was used to perform all the meta-analyses.

### Sensitivity analyses

To assess the influences of modeling and analytical choices as well as the effects of interindividual variability of SC and noise, we performed a series of sensitivity analyses on a randomly selected subsample of 200 subjects (stratified by sex and age bin) from the PNC dataset and compared them to the main run.

#### Interindividual variability of the structural connectome

We assessed the potential effect of the interindividual variability of SCs in our findings by performing the following analyses: (i) We studied the effect of age on the E-I ratio, additionally controlling for the SC strength of each node, calculated as the row-wise sum of the SC. (ii) We performed BNM simulation-optimization using subject-specific functional data as the target but with the template SC of the MICs dataset determining the connectivity of model nodes, thereby eliminating the interindividual variability of SCs as a potential source of variability in the regional E-I ratio. These higher-quality DWI data from an adult sample were chosen to additionally assess the robustness of our results to potential inaccuracies of subject-level SCs derived from relatively lower-quality DWI data of the adolescent datasets.

#### Parcellation, heterogeneity of regional parameters, interhemispheric connections, and Gaussian noise seed

In these sensitivity analyses, we performed the BNM simulation-optimization using alternative modeling configurations, including (i) using the Schaefer-200 parcellation, (ii) using T1w/T2w and FC G1 as the heterogeneity maps, (iii) using NMDA and GABA_A/BZ_ as the heterogeneity maps, (iv) using T1w/T2w, FC G1, NMDA, and GABA_A/BZ_ as the heterogeneity maps, (v) using six null maps generated by randomly spinning the original maps together, as implemented in the ENIGMA Toolbox (*126*), (vi) assuming homogeneous regional parameters $w_{i}^{\text{EE}}$ and $w_{i}^{\text{EI}}$ in a three-parameter “homogeneous” model, (vii) assigning independent free parameters for each regional parameter of each node in a 201-parameter “node-independent” model, (viii) including interhemispheric connections in the goodness of fit and cost calculations, and (ix) using a different Gaussian noise seed than the default. Subsequently, we compared these optimal simulations derived from these alternative models with the main run in terms of goodness-of-fit measures, test-retest reliability of E-I ratio, and the effect of age on the E-I ratio. To assess the effect of Gaussian noise seed on the findings, we additionally calculated the effect of age on the E-I ratio based on average estimates from models using the default and alternative noise seeds. Furthermore, in the “node-based heterogeneous” model, we assessed the level of optimizer convergence based on the range (max – min) of goodness of fit across the particles in the last generation and compared it to that of the main run using paired *t* test.

#### Effects of Gaussian noise seed and interregional conduction delay in optimal simulations of the main run

These analyses were performed by repeating the optimal simulation of each subject obtained in the main analyses with alternative configurations, including (i) using 50 different randomization seeds for generating the Gaussian noise injected into the model (Eqs. 5 and 6) and (ii) adding conduction delay in the signal transmission between the model nodes. For the latter, delay was calculated as the SC edge length obtained from the tractography of each subject, divided by a conduction velocity. For each subject, we performed six alternative simulations with variable conduction velocities in the range of {1, …, 6} m/s. Of note, in simulations with conduction delay, a recent history of $S_{i}^{E}$ in all nodes needs to be stored in GPU memory so that the input of node *j* to node *i* at time *t* can be determined on the basis of $S_{j}^{E}$ at ${\text{delay}}_{\mathrm{ij}}$ time points ago. Accordingly, to reduce memory needed for storing this history, we performed these simulations by updating the global input to each node at intervals of 1 ms, instead of 0.1 ms used in the main analyses. Subsequently, we calculated the goodness-of-fit measures of the alternative simulations to the empirical data of each subject and compared them with the goodness-of-fit measures of the main simulation. In addition, we calculated the ICC of E-I ratio between the main simulation and each of the alternative simulations. In the case of Gaussian noise seeds, we combined the goodness of fit as well as ICC measures of the 50 different seeds by taking their median.

#### Ground truth recovery analysis

We first generated synthetic functional data by running a ground truth simulation with known (and arbitrary) model parameters and using the template SC. Then, similar to the approach used for fitting the models to the real empirical data of subjects, we performed two BNM simulation-optimization runs aimed to maximize the model fit to the synthetic FC and FCD of the ground truth simulation. Next, we selected the best of the optima resulting from two runs as the “recovered optimal simulation” and compared it to the ground truth simulation in terms of goodness-of-fit measures and recovery of regional E-I ratio. For assessing the recovery of regional E-I ratio, we used Pearson correlation and ICC to compare the arrays of recovered versus ground truth simulations. We performed two alternative types of recovery optimization runs: (i) using the same Gaussian noise seed as the ground truth simulation or (ii) using 50 alternative Gaussian noise seeds that differed from the one used in the ground truth simulation.

## Supplementary Materials

### This PDF file includes:

List of IMAGEN Consortium members Figs. S1 to S21 Table S1
