# Supplementary material for: Adolescent maturation of cortical excitation-inhibition ratio based on individualized biophysical network modeling
Source: Sci Adv. 2025 Jun 4;11(23):eadr8164. doi: 10.1126/sciadv.adr8164 (PMC12136046; doi:10.1126/sciadv.adr8164)
Supplement: Supplementary file 1 — List of IMAGEN Consortium members Figs. S1 to S21 Table S1 [file sciadv.adr8164_sm.pdf]

Science Advances
AAAS

Supplementary Materials for

Adolescent maturation of cortical excitation-inhibition ratio based on individualized biophysical network modeling

Amin Saberi *et al.*

Corresponding author: Sofie L. Valk, s.valk@fz-juelich.de

Sci. Adv. **11**, eadr8164 (2025)
DOI: 10.1126/sciadv.adr8164

This PDF file includes:

List of IMAGEN Consortium members
Figs. S1 to S21
Table S1

IMAGEN Consortium Members and Affiliations

Tobias Banaschewski[1], Gareth J. Barker[2], Arun L.W. Bokde[3], Sylvane Desrivières[4], Herta Flor[5,6], Antoine Grigis[7], Hugh Garavan[8], Penny Gowland[9], Andreas Heinz[10,11], Rüdiger Brühl[12], Jean-Luc Martinot[13], Marie-Laure Paillère Martinot[13,14], Eric Artiges[13,15], Frauke Nees[1,5,16], Dimitri Papadopoulos Orfanos[7], Herve Lemaitre[7,17], Luise Poustka[18], Sarah Hohmann[1], Nathalie Holz[1], Christian Baeuchl[19], Michael N. Smolka[19], Nilakshi Vaidya[20], Henrik Walter[10,11], Robert Whelan[21], Gunter Schumann[20,22,23,24], Tomáš Paus[25]

[1] Department of Child and Adolescent Psychiatry and Psychotherapy, Central Institute of Mental Health, Medical Faculty Mannheim, Heidelberg University, Square J5, 68159 Mannheim, Germany;
[2] Department of Neuroimaging, Institute of Psychiatry, Psychology & Neuroscience, King's College London, United Kingdom;
[3] Discipline of Psychiatry, School of Medicine and Trinity College Institute of Neuroscience, Trinity College Dublin, Dublin, Ireland;
[4] Social, Genetic and Developmental Psychiatry Centre, Institute of Psychiatry, Psychology & Neuroscience, King's College London, United Kingdom;
[5] Institute of Cognitive and Clinical Neuroscience, Central Institute of Mental Health, Medical Faculty Mannheim, Heidelberg University, Square J5, Mannheim, Germany;
[6] Department of Psychology, School of Social Sciences, University of Mannheim, 68131 Mannheim, Germany;
[7] NeuroSpin, CEA, Université Paris-Saclay, F-91191 Gif-sur-Yvette, France;
[8] Departments of Psychiatry and Psychology, University of Vermont, 05405 Burlington, Vermont, USA;
[9] Sir Peter Mansfield Imaging Centre School of Physics and Astronomy, University of Nottingham, University Park, Nottingham, United Kingdom;
[10] Department of Psychiatry and Psychotherapy CCM, Charité – Universitätsmedizin Berlin, corporate member of Freie Universität Berlin, Humboldt-Universität zu Berlin, and Berlin Institute of Health, Berlin, Germany;
[11] German Center for Mental Health (DZPG), site Berlin-Potsdam, Germany;
[12] Physikalisch-Technische Bundesanstalt (PTB), Braunschweig and Berlin, Germany;
[13] Institut National de la Santé et de la Recherche Médicale, INSERM U1299 "Trajectoires développementales en psychiatrie"; Université Paris-Saclay, Ecole Normale supérieure Paris-Saclay, CNRS, Centre Borelli; Gif-sur-Yvette, France;
[14] AP-HP. Sorbonne Université, Department of Child and Adolescent Psychiatry, Pitié-Salpêtrière Hospital, Paris, France;
[15] Psychiatry Department, EPS Barthélémy Durand, Etampes, France;
[16] Institute of Medical Psychology and Medical Sociology, University Medical Center Schleswig-Holstein, Kiel University, Kiel, Germany;
[17] Institut des Maladies Neurodégénératives, UMR 5293, CNRS, CEA, Université de Bordeaux, 33076 Bordeaux, France;
[18] Department of Child and Adolescent Psychiatry, Center for Psychosocial Medicine, University Hospital Heidelberg, Heidelberg, Germany;
[19] Department of Psychiatry and Psychotherapy, Technische Universität Dresden, Dresden, Germany;
[20] Centre for Population Neuroscience and Stratified Medicine (PONS), Department of Psychiatry and Psychotherapy, Charité Universitätsmedizin Berlin, Germany;
[21] School of Psychology and Global Brain Health Institute, Trinity College Dublin, Ireland;
[22] Centre for Population Neuroscience and Precision Medicine (PONS), Institute for Science and Technology of Brain-inspired Intelligence (ISTBI), Fudan University, Shanghai, China.
[23] Department of Psychiatry, University of Cambridge, UK.
[24] German Centre for Mental Health.
[25] Departments of Psychiatry and Neuroscience, Faculty of Medicine and Centre Hospitalier Universitaire Sainte-Justine, University of Montreal, Montreal, Quebec, Canada;

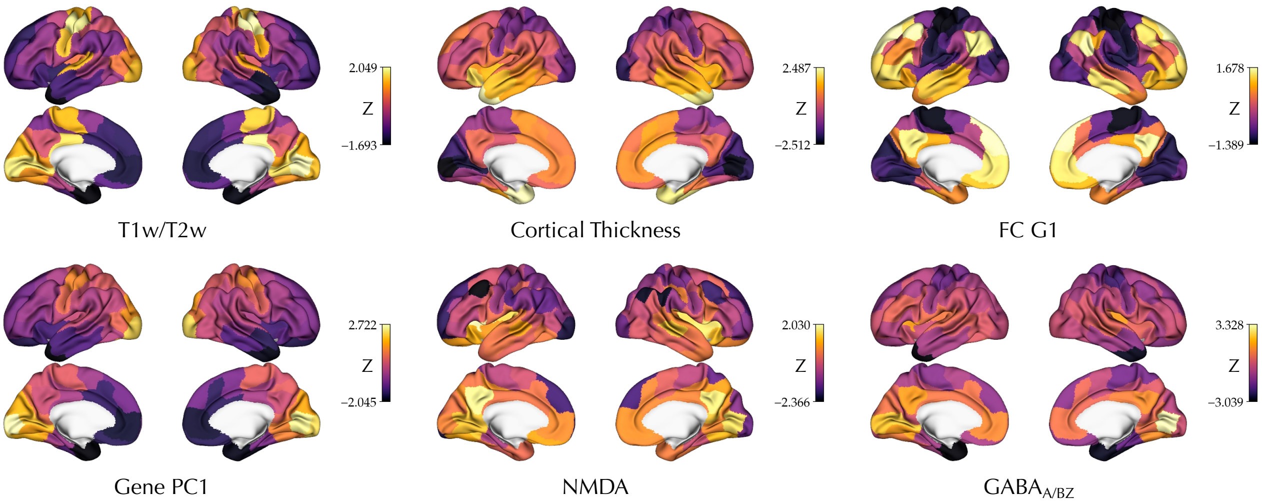

Fig. S1. Biological heterogeneity maps. The Z-scored maps shown were used to determine regional heterogeneity of the local recurrent excitatory w_i^{EE} and excitatory-to-inhibitory w_i^{EI} connectivity weights within the simulations.

T1w/T2w: T1-weighted to T2-weighted ratio, FC G1: principal gradient of functional connectivity, Gene PC1: principal axis of Allen Human Brain Atlas gene expression data, NMDA: N-methyl-D-aspartate receptor density, $GABA_{A/BZ}$: γ-aminobutyric acid type A/Bz receptor density.

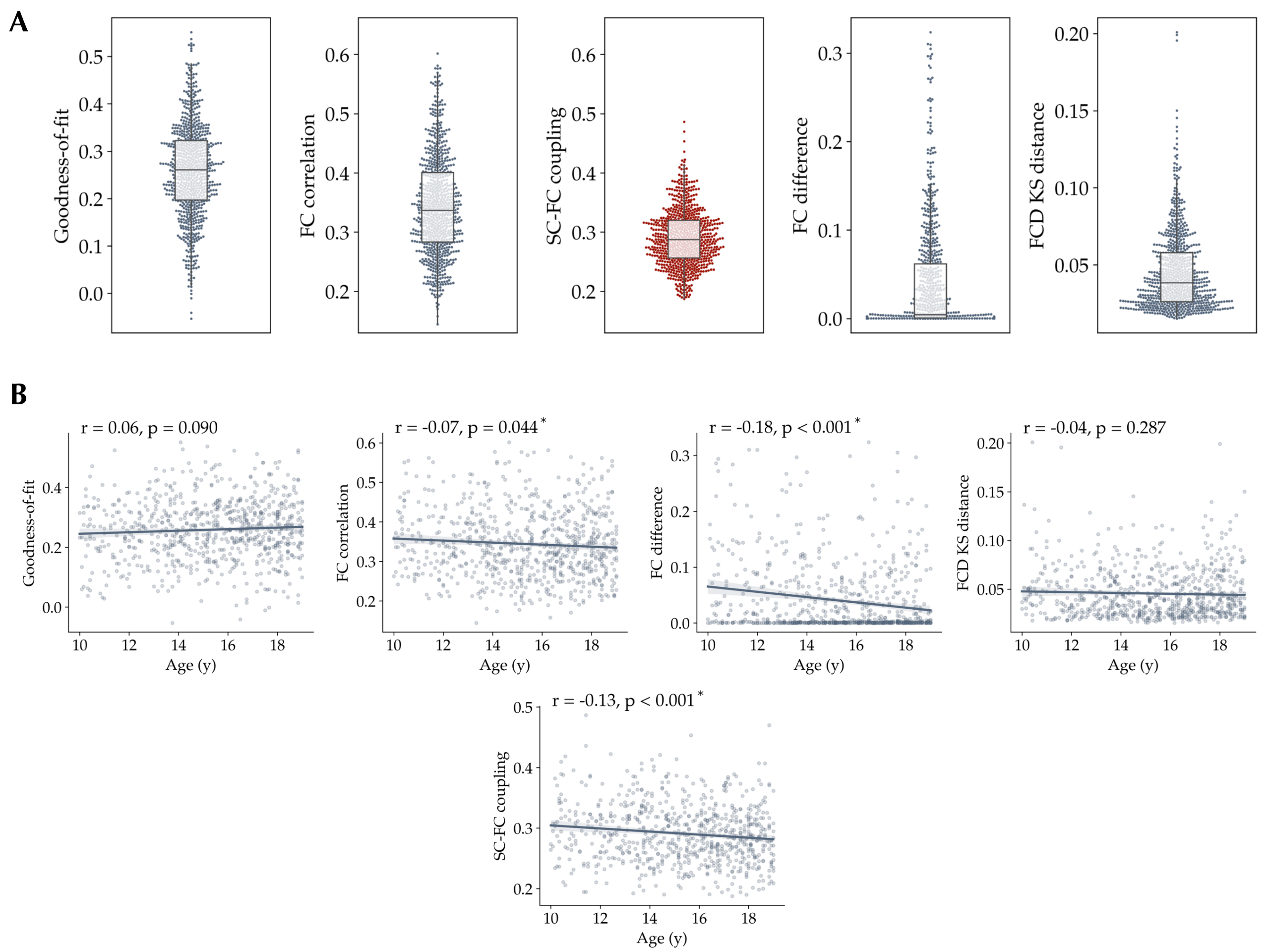

Fig. S2. Goodness-of-fit measures in the PNC dataset. (A) Distribution of the goodness-of-fit measures. The coupling of the structural connectome (SC) and the empirical functional connectome (FC) is independent of the simulations, and is shown as a reference for FC correlation of simulated and empirical data. **(B)** Pearson correlation between the goodness-of-fit measures and age.
FCD: functional connectivity dynamics matrix, KS: Kolmogorov-Smirnov distance.

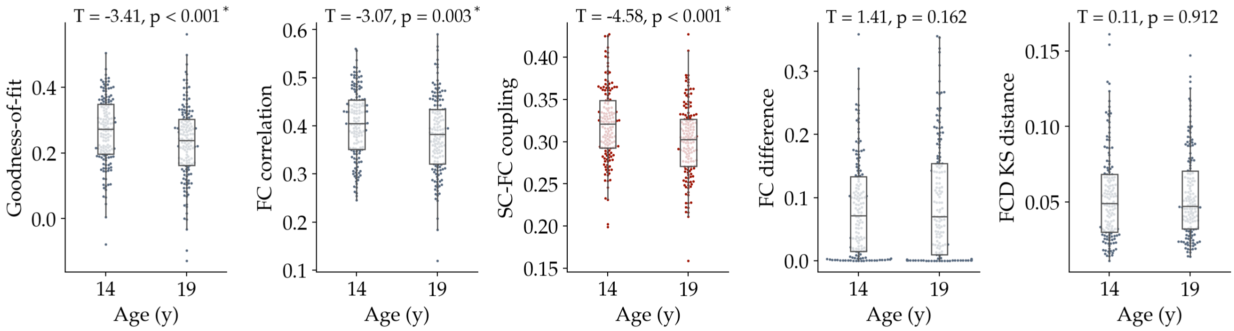

Fig. S3. Goodness-of-fit measures in the IMAGEN dataset. Distribution of goodness-of-fit measures in the baseline (14 y) and follow-up (19 y) imaging sessions is shown. The goodness-of-fit measures were compared between the two sessions using paired T-tests. The coupling of the structural connectome (SC) and the empirical functional connectome (FC) is independent of the simulations, and is shown as a reference for FC correlation of simulated and empirical data.

FCD: functional connectivity dynamics matrix, KS: Kolmogorov-Smirnov distance.

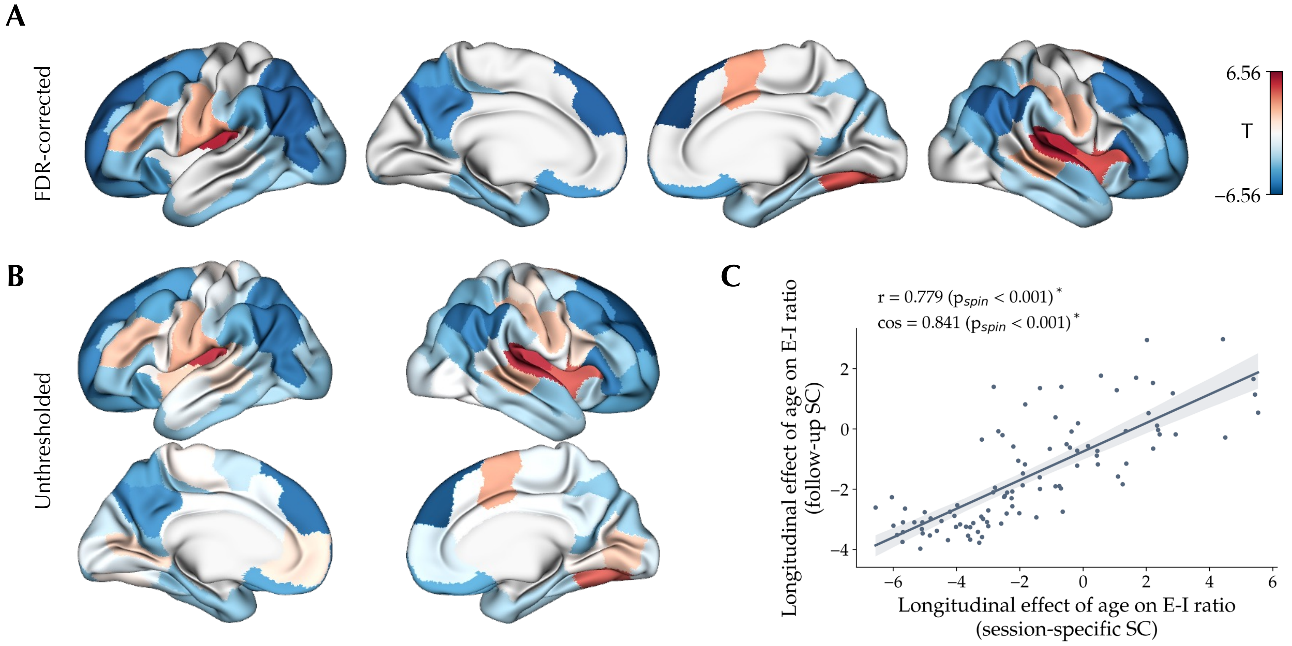

Fig. S4. Longitudinal effect of age on E-I ratio during adolescence based on session-specific structural connectomes. (A) Longitudinal effect of age on E-I ratio in a mixed effects model with random intercepts for each subject, after removing outliers and controlling for goodness-of-fit, sex, in-scanner rs-fMRI motion and site, corrected for multiple comparisons using false discovery rate (FDR). **(B)** The unthresholded effect of age on E-I ratio. **(C)** Spatial co-alignment (Pearson correlation [r] or cosine similarity [cos]) of longitudinal effects of age on E-I ratio using session-specific structural connectomes (SCs) compared with the age effects when the SC of the follow-up session was used in the modeling of both sessions.

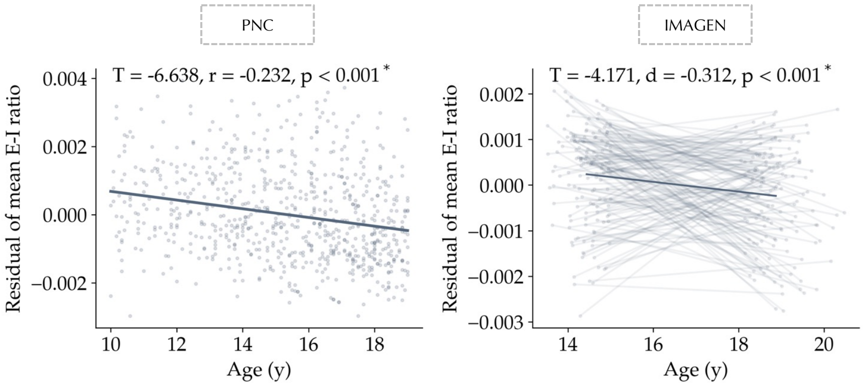

Fig. S5. Effect of age on E-I ratio across the conjunction mask of significant age effects across datasets. The effect of age on the mean E-I ratio across 33 regions showing significant replicable decreases of the E-I ratio in the PNC and IMAGEN datasets (Fig. 3D) was investigated, controlling for sex, goodness-of-fit, in-scanner rs-fMRI motion, and in IMAGEN, site. Points in both plots represent the residual of the mean E-I ratio within the conjunction mask after removing confounds for each individual subject and session. The thick line in PNC shows best linear fit and in IMAGEN connects the averages of the two sessions across all subjects. Thin lines in IMAGEN show longitudinal changes of this measure during follow-up in each subject.

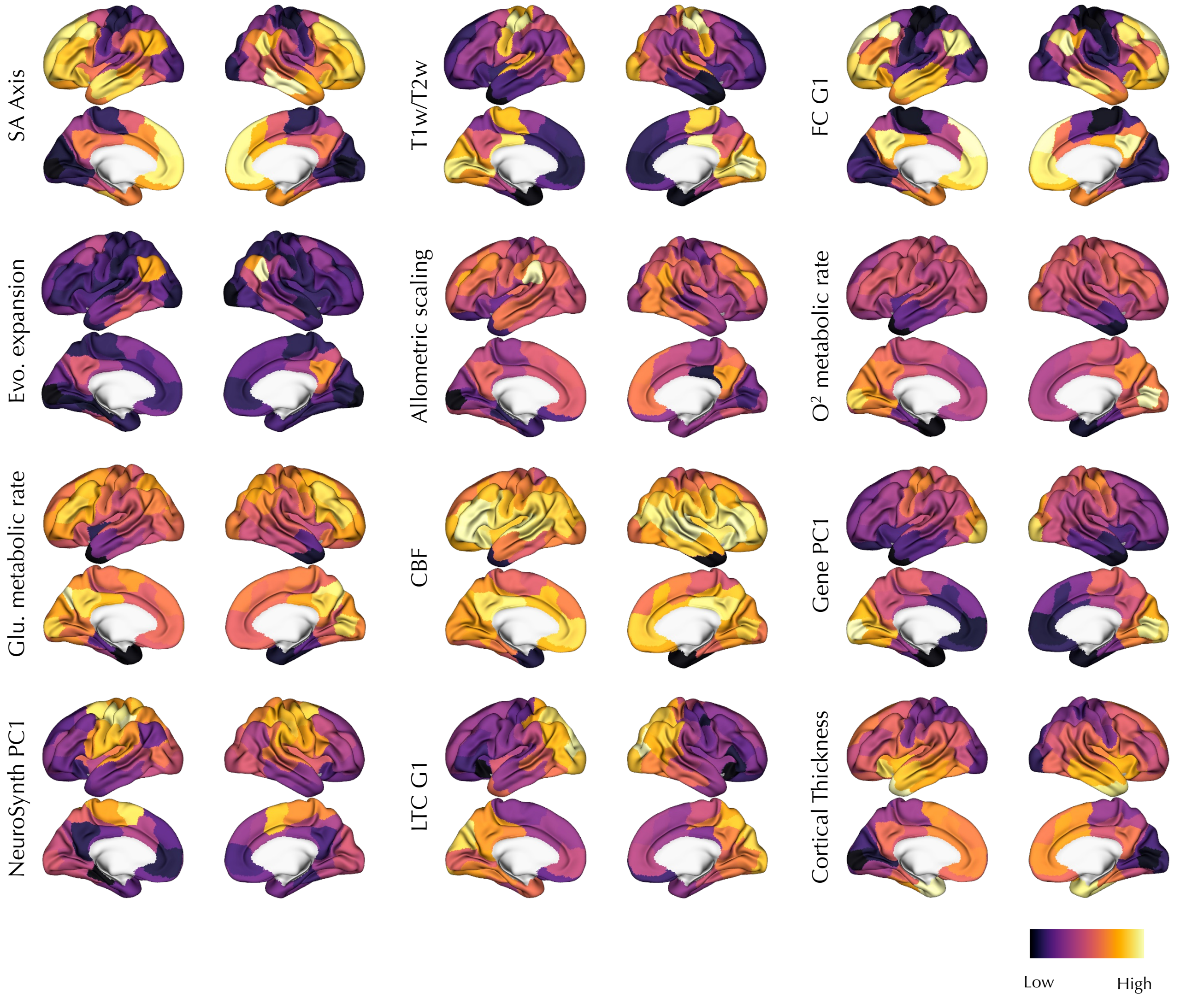

Fig. S6. Multimodal maps of the sensorimotor-association cortical axis. The map of the sensorimotor-association (SA) axis proposed in Sydnor et al. (18) as well as its components or their substitutes is shown. See Table S1 for the sources of each map.

T1w/T2w: T1-weighted to T2-weighted ratio, FC G1: principal gradient of functional connectivity, Evo.: evolutionary, CMR: cerebral metabolic rate, Glu.: glucose, CBF: cerebral blood flow, Gene PC1: principal axis of Allen Human Brain Atlas gene expression data, NeuroSynth PC1: Principal component of NeuroSynth meta-analytical maps, LTC G1: principal gradient of laminar thickness covariance.

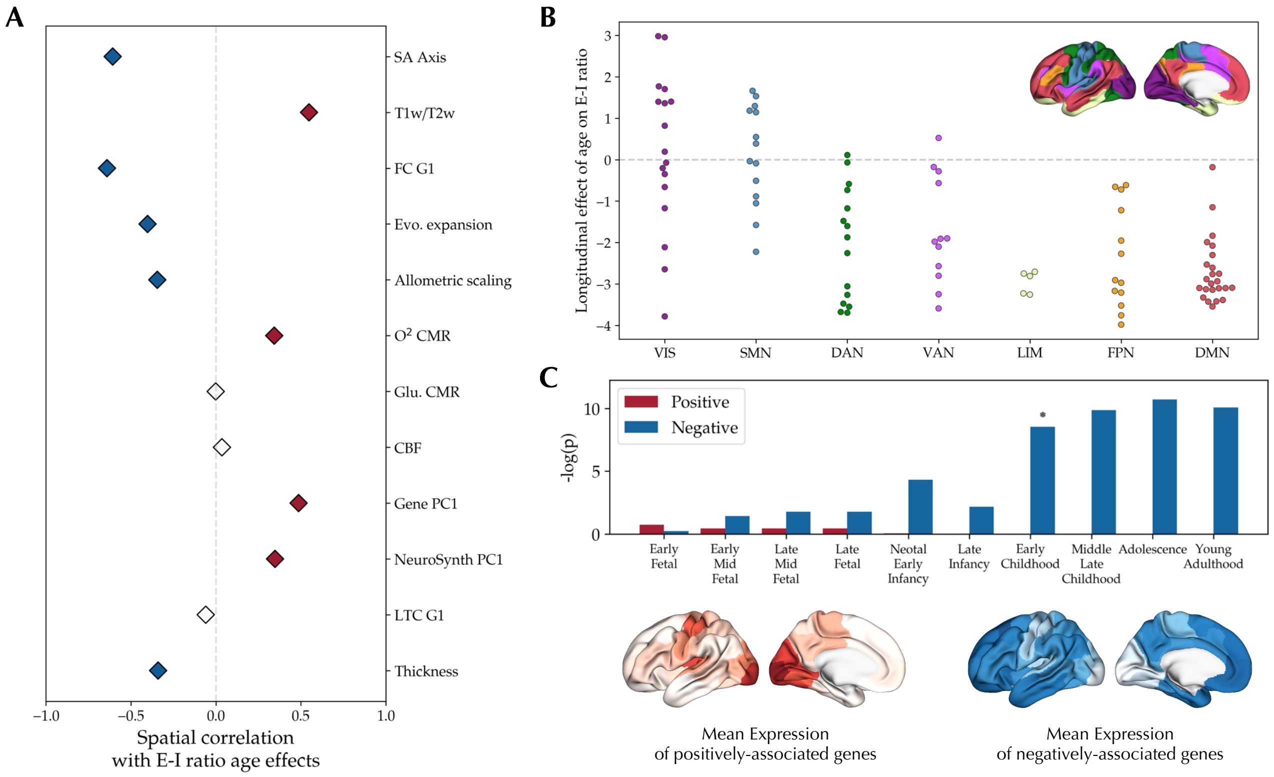

Fig. S7. Embedding of the E-I ratio developmental pattern in the IMAGEN dataset along the sensorimotor-association axis. (**A**) Spatial correlation of the E-I ratio maturation map in the IMAGEN dataset with the maps of sensorimotor-association cortical axis based on Sydnor et al. (18) (Fig. S6). Colored diamonds show statistically significant ($p_{spin} < 0.05$) positive (red) and negative (blue) spatial correlations. **(B)** Distribution of the E-I ratio maturation map across the canonical resting-state networks ($F = 14.57$, $p_{spin} < 0.001$). Post-hoc tests (Bonferroni-corrected) showed significantly more positive age effects in the visual (VIS) and somatomotor (SMN) compared to the limbic (LIM), dorsal attention (DAN) and default mode networks (DMN) in addition to more positive age effects in SMN compared to the ventral attention (VAN) and frontoparietal (FPN) networks. **(C)** *Bottom*: Mean expression of the top 500 genes associated with the E-I ratio maturation map, split into sets of negatively-associated (N = 216, blue) and positively-associated (N = 284, red) genes. *Top:* Specific expression analysis of the two sets of genes across developmental stages in the cortex. Y-axis shows the negative log of false discovery rate (FDR)-corrected p-values. Asterisks denote significantly enriched developmental stages compared to null genes based on spin surrogate maps (1000 permutations) and after FDR adjustment.

SA: sensorimotor-association, T1w/T2w: T1-weighted to T2-weighted ratio, FC G1: principal gradient of functional connectivity, Evo.: evolutionary, CMR: cerebral metabolic rate, Glu.: glucose, CBF: cerebral blood flow, Gene PC1: principal axis of Allen Human Brain Atlas gene expression data, NeuroSynth PC1: Principal component of NeuroSynth meta-analytical maps, LTC G1: principal gradient of laminar thickness covariance.

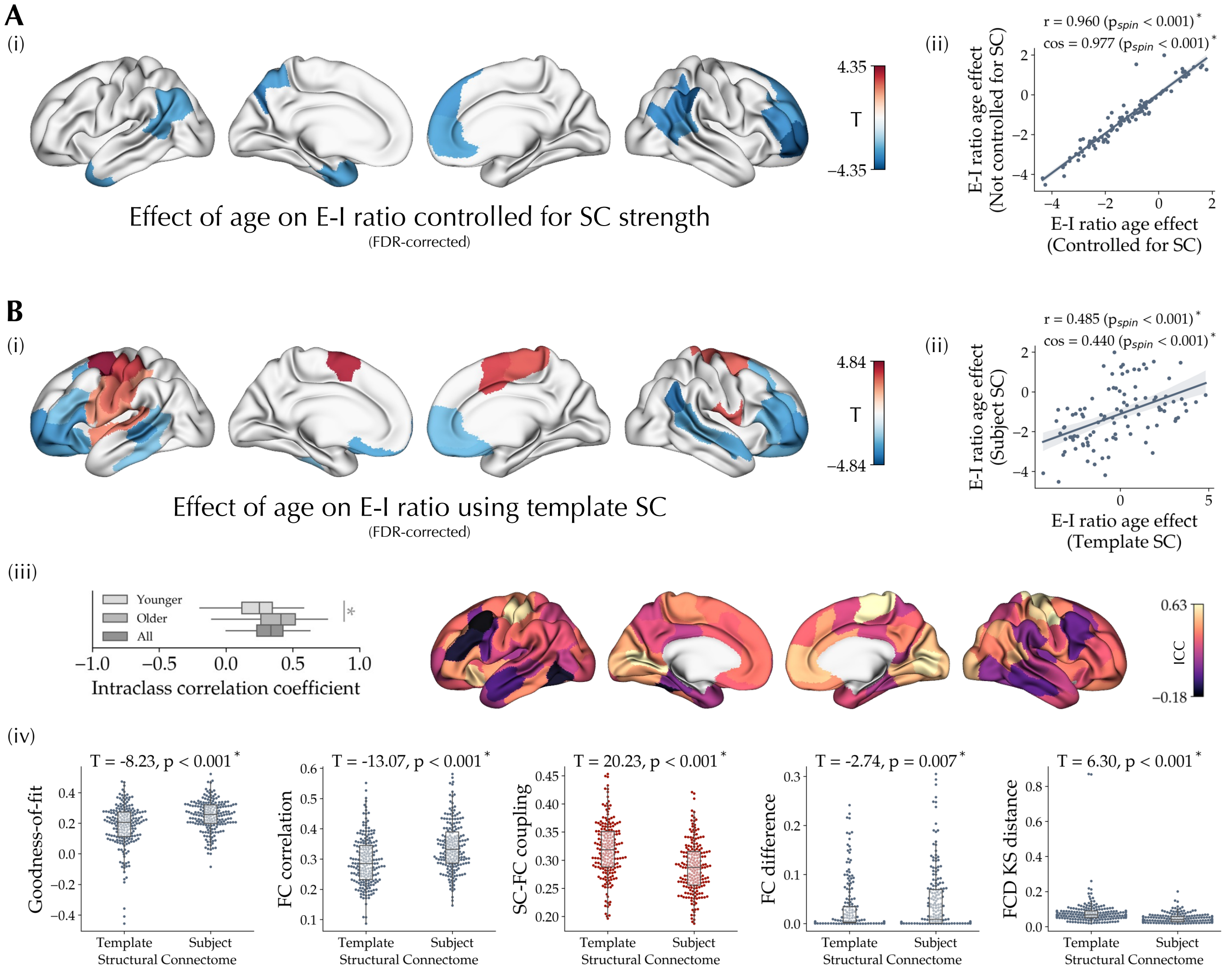

Fig. S8. Robustness of the findings to the effect of inter-individual variability of structural connectome. (A) *(i)* Effect of age on E-I ratio in the PNC subsample (N = 200) controlled for age, sex, goodness-of-fit, and in-scanner motion, in addition to node-wise structural connectome (SC) strength (i.e., the row-wise sum of the SC matrix), and after false discovery rate (FDR) correction. *(ii):* Spatial co-alignment (Pearson correlation [r] or cosine similarity [cos]) of the unthresholded effect of age on E-I ratio with and without controlling for SC strength. **(B)** *(i)*: Effect of age on E-I ratio in the PNC subsample using a fixed template SC in the models, after FDR correction. *(ii):* Spatial co-alignment of the unthresholded effect of age on E-I ratio based on models using subject-specific versus template SCs. *(iii)*: Distribution and map of the node-wise median absolute deviation intraclass correlation coefficient (ICC) of E-I ratio between the main model, using subject-specific SC, and this model, using template SC, calculated across all subsample subjects (dark gray; also shown on the map), older half (medium gray), or younger half (light gray). Asterisk denotes significant paired T-test comparing ICCs of younger and older halves across nodes. *(iv):* Comparison of goodness-of-fit measures and coupling of SC and empirical functional connectome (FC) between models based on subject-specific versus template SCs. T- and p-values resulting from paired T-tests are reported.

FCD: functional connectivity dynamics matrix, KS: Kolmogorov-Smirnov distance.

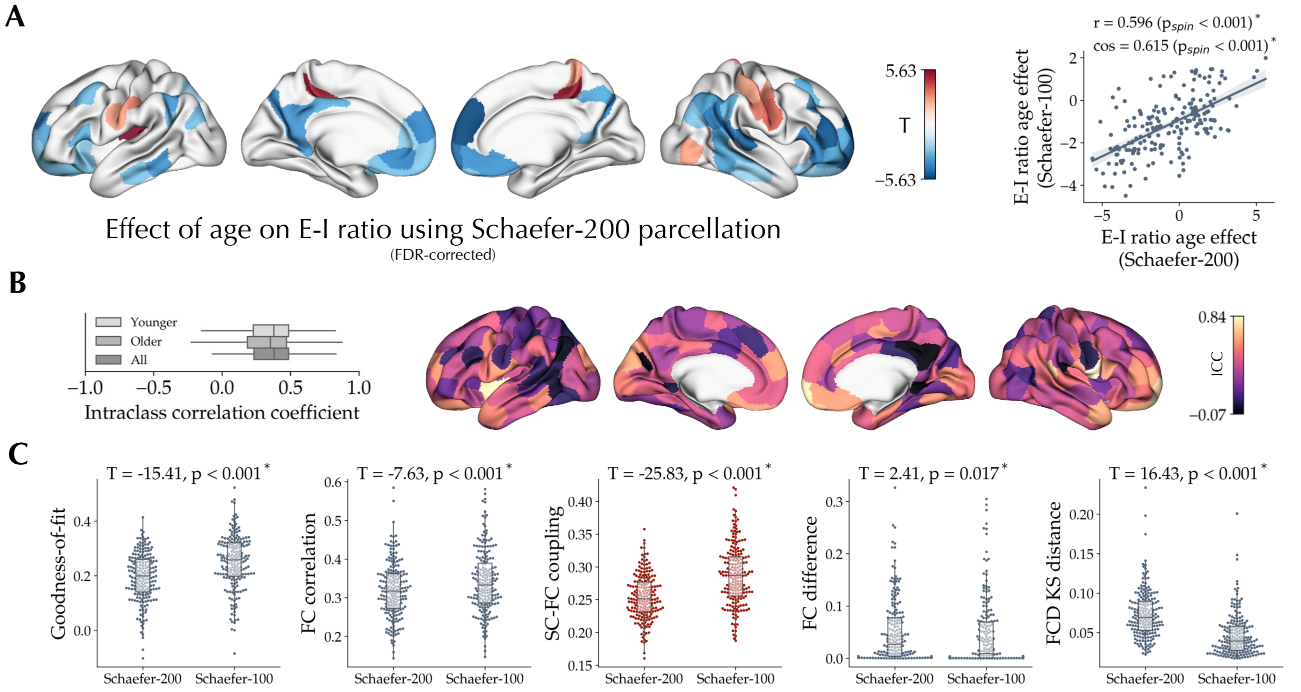

Fig. S9. Robustness of the findings to the effect of parcellation. (A) *Left*: Effect of age on E-I ratio in the PNC subsample (N = 200) using Schaefer-200 parcellation, after false discovery rate (FDR) correction. *Right:* Spatial co-alignment (Pearson correlation [r] or cosine similarity [cos]) of the unthresholded effect of age on E-I ratio between models using Schaefer-100 and Schaefer-200 parcellations. **(B)** Distribution and map of the node-wise median absolute deviation intraclass correlation coefficient (ICC) of E-I ratio between the main model (using Schaefer-100; upsampled to Schaefer-200) and the model using Schaefer-200, calculated across all subsample subjects (dark gray; also shown on the map), older half (medium gray), or younger half (light gray). Paired T-test comparing ICCs of younger and older halves across nodes showed no significant difference. **(C)** Comparison of goodness-of-fit measures and coupling of structural connectome (SC) and empirical functional connectome (FC) between models based on Schaefer-100 and Schaefer-200 parcellations. T- and p-values resulting from paired T-tests are reported.

FCD: functional connectivity dynamics matrix, KS: Kolmogorov-Smirnov distance.

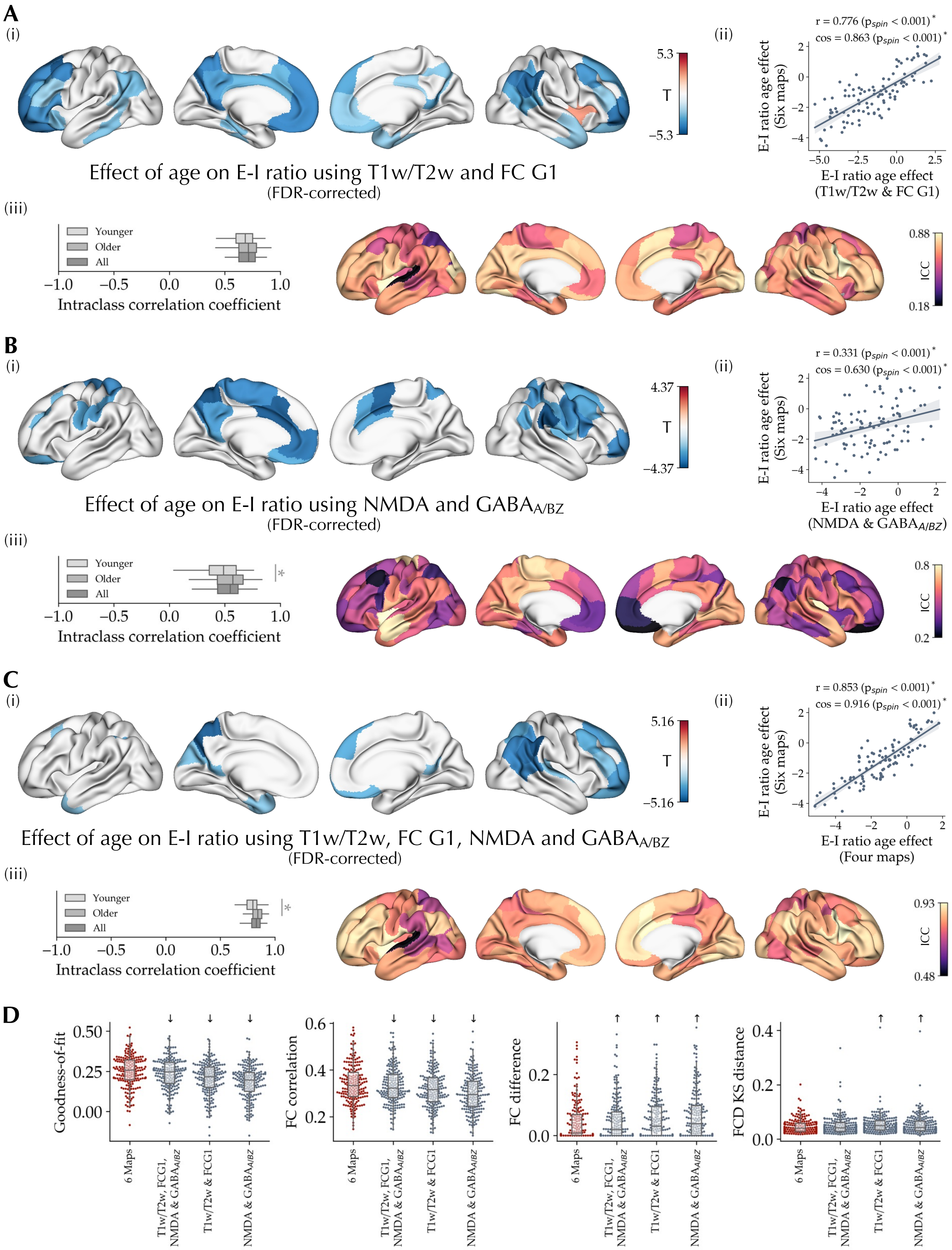

Fig. S10. Robustness of the findings to the effect of heterogeneity maps. (A-C) *(i)*: Effect of age on E-I ratio in the PNC subsample (N = 200) using alternative sets of heterogeneity maps, after false discovery rate (FDR) correction. *(ii):* Spatial co-alignment (Pearson correlation [r] or cosine similarity [cos]) of the unthresholded effect of age on E-I ratio between the models using alternative sets of maps and the main model, which uses all the six heterogeneity maps. *(iii)*: Distribution and map of the node-wise median absolute deviation intraclass correlation coefficient (ICC) of E-I ratio between the main model (using all the six heterogeneity maps) and the model using alternative sets of maps, calculated across all subsample subjects (dark gray; also shown on the maps), older half (medium gray), or younger half (light gray). Asterisks in **B** and **C** denote significant paired T-tests comparing

ICCs of younger and older halves across nodes. **(D)** Comparison of goodness-of-fit measures between models based on two, four and six heterogeneity maps. Down-/upward arrows indicate significant (paired T-test $p < 0.05$) de-/increases of the goodness-of-fit measures in these models compared to the main model.

T1w/T2w: T1-weighted to T2-weighted ratio, FC G1: principal gradient of functional connectivity, NMDA: N-methyl-D-aspartate receptor density, $GABA_{A/BZ}$: γ-aminobutyric acid type A/Bz receptor density, FC: functional connectivity, FCD: functional connectivity dynamics matrix, KS: Kolmogorov-Smirnov distance.

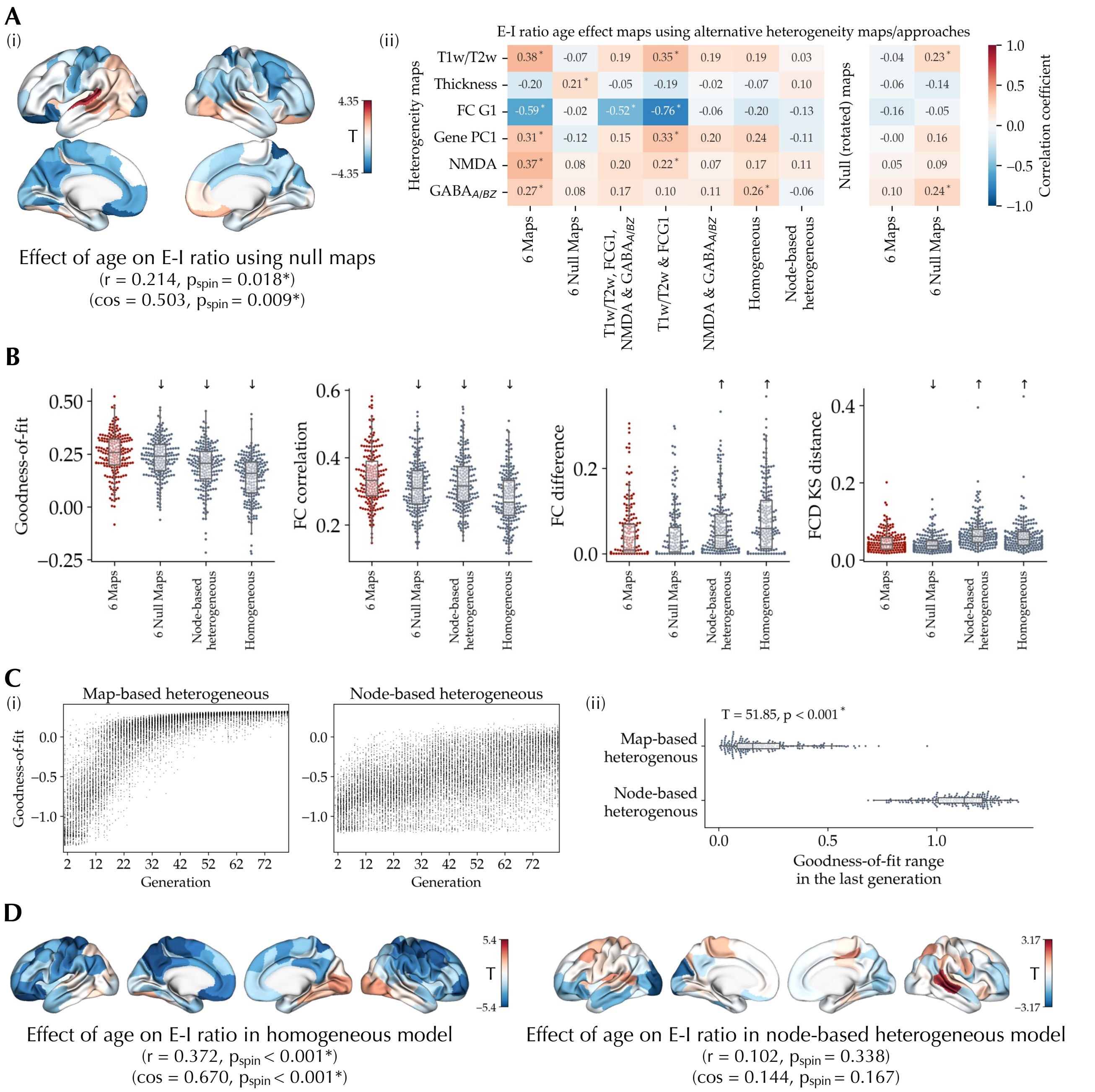

Fig. S11. Comparison of models based on true, null, or no heterogeneity maps. (A) *(i)*: The unthresholded effect of age on E-I ratio in the PNC subsample (N = 200) is calculated based on a model in which a set of six null maps (created by randomly spinning the original maps) determined heterogeneity of regional parameters. Spatial co-alignment (Pearson correlation [r] or cosine similarity [cos]) of the unthresholded effect of age on E-I ratio between this model and the main model that was based on the true maps, is reported. *(ii):* Spatial correlation of the true or null heterogeneity maps (rows) with the unthresholded maps of effect of age on E-I ratio (columns) observed across models using alternative approaches for determining heterogeneity (or lack thereof) of regional parameters. Asterisks denote significant correlations in the spin test. **(B)** Comparison of goodness-of-fit measures between the main model based on six true maps (red) and the model based on null maps, as well as the 'node-based heterogeneous' and 'homogeneous' models, which were map-free. Down-/upward arrows indicate significant (paired T-test $p < 0.05$) de-/increases of the goodness-of-fit measures in these models compared to the main model. **(C)** *(i)*: The evolution of goodness-of-fit in particles (dots) sampled through generations of the covariance matrix adaptation-evolution strategy (CMA-ES) in the map-based (main) and node-based heterogeneous models for an example subject. *(ii)*: Across subsample subjects, the range (difference between maximum and minimum) of goodness-of-fit across particles of the last CMA-ES generation, was significantly higher in the node-based heterogeneous compared to the map-based (main) model, indicating the lower rate of convergence in the node-based heterogeneous model. **(D)** The unthresholded effect of age on E-I ratio in the PNC subsample (N = 200) based on the homogeneous (*left*) and node-based heterogeneous (*right*) models is shown.

Spatial co-alignment of the unthresholded effect of age on E-I ratio between these models and the main model is reported.

T1w/T2w: T1-weighted to T2-weighted ratio, FC G1: principal gradient of functional connectivity, Gene PC1: principal axis of Allen Human Brain Atlas gene expression data, NMDA: N-methyl-D-aspartate receptor density, $GABA_{A/BZ}$: γ-aminobutyric acid type A/Bz receptor density, FC: functional connectivity, FCD: functional connectivity dynamics matrix, KS: Kolmogorov-Smirnov distance.

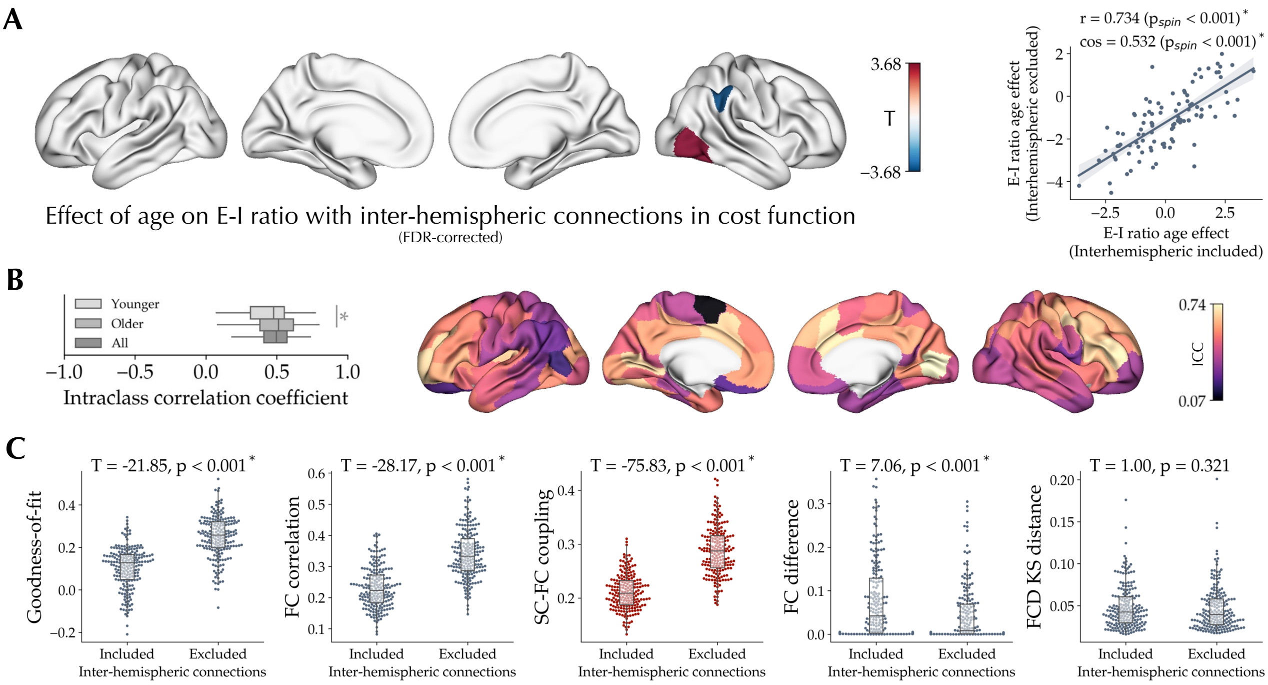

Fig. S12. Robustness of the findings to the inclusion of inter-hemispheric connections in the cost function. (A) *Left*: Effect of age on E-I ratio in the PNC subsample (N = 200) with inter-hemispheric connections included in the cost function, after false discovery rate (FDR) correction. *Right:* Spatial co-alignment (Pearson correlation [r] or cosine similarity [cos]) of the unthresholded effect of age on E-I ratio between models with the inter-hemispheric connections included or excluded from the cost function. **(B)** Distribution and map of the node-wise median absolute deviation intraclass correlation coefficient (ICC) of E-I ratio between the models with or without inter-hemispheric connections included in the cost, calculated across all subsample subjects (dark gray; also shown on the map), older half (medium gray), or younger half (light gray). Asterisk denotes significant paired T-test comparing ICCs of younger and older halves across nodes. **(C)** Comparison of goodness-of-fit measures and coupling of structural connectome (SC) and empirical functional connectome (FC) between models fit with the inter-hemispheric connections included or excluded from the cost function. T- and p-values resulting from paired T-tests are reported.

FCD: functional connectivity dynamics matrix, KS: Kolmogorov-Smirnov distance.

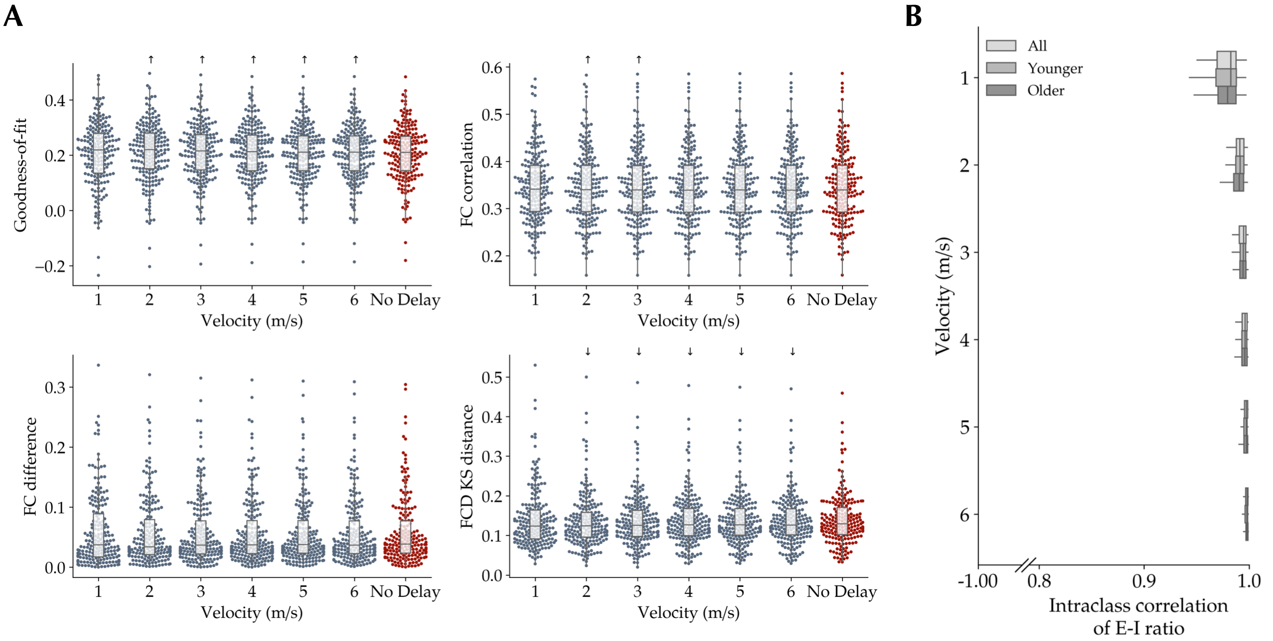

Fig. S13. Effect of conduction delay on goodness-of-fit and E-I ratio. (A) Variation of goodness-of-fit and its components as a function of conduction velocity in the optimal simulations of the PNC subsample (N = 200). The delayed-conduction simulations showed small but significant differences (↑: increase, ↓: decrease) in goodness-of-fit measures compared to the main model without delay (red). The mean difference of goodness-of-fit ranged from -0.0005 using a velocity of 1 m/s to 0.004 using a velocity of 2 m/s. **(B)** Distribution of the median absolute deviation intraclass correlation coefficient (ICC) of E-I ratio between non-delayed-conduction and delayed-conduction simulations across nodes as a function of conduction velocity, calculated across all subsample subjects (dark gray), older half (medium gray), or younger half (light gray). Paired T-tests comparing ICCs of younger and older halves across nodes showed no significant differences.

FC: functional connectivity, FCD: functional connectivity dynamics matrix, KS: Kolmogorov-Smirnov distance.

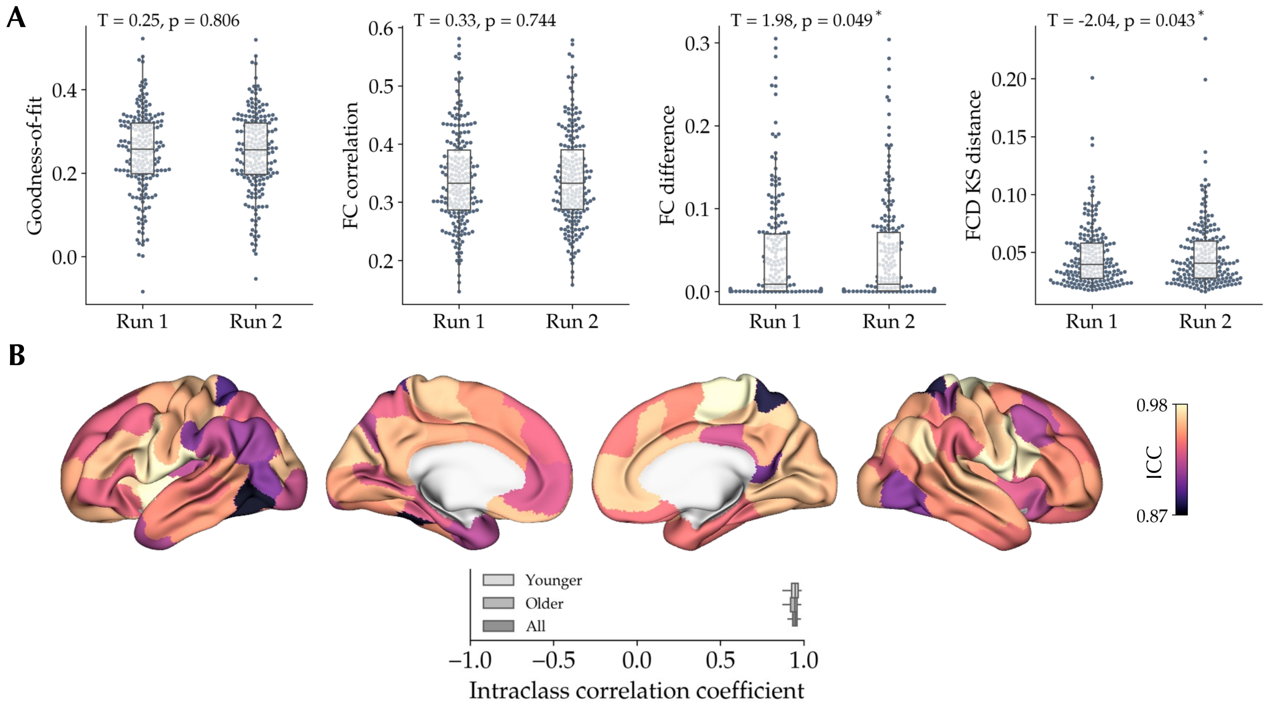

Fig. S14. Effect of optimization seeds on goodness-of-fit and E-I ratio. (A) Comparison of goodness-of-fit measures between the two optimization runs with different random seeds in the PNC subsample (N = 200). **(B)** Node-wise median absolute deviation intraclass correlation coefficient (ICC) of E-I ratio between the two runs, calculated across all subsample subjects (dark gray; also shown on the map), older half (medium gray), or younger half (light gray). Paired T-test comparing ICCs of younger and older halves across nodes showed no significant difference.

FC: functional connectivity, FCD: functional connectivity dynamics matrix, KS: Kolmogorov-Smirnov distance.

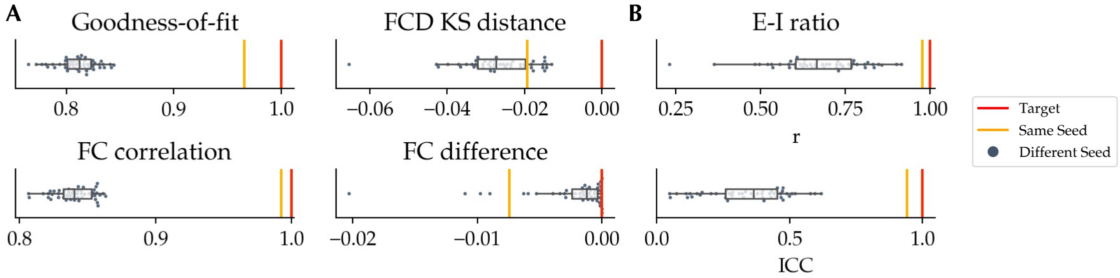

Fig. S15. Ground truth recovery analysis. The optimal simulations that were fitted to ground truth synthetic functional data (with known parameters) using two runs of covariance matrix adaptation-evolution strategy were compared to the ground truth in terms of goodness-of-fit measures (including functional connectivity [FC] correlation, FC mean absolute difference, FC dynamics [FCD] Kolmogorov-Smirnov [KS] distance; Panel **A**), and regional E-I ratio ($\langle I_i^E \rangle$; Panel **B**). The recovery simulation-optimization runs were performed either using the same simulation seed (orange vertical lines) as the ground truth simulation, or different simulation seeds (scatter points and box plot). The targets (red vertical lines) of the recovery were for the resulting optimal simulations (across two recovery runs per simulation seed) to show perfect goodness-of-fit, and high alignment of E-I ratio maps with that of the ground truth simulation. Regional values of E-I ratio were compared between the recovered optimal simulations and the ground truth using Pearson correlation (r) and median absolute deviation intraclass correlation coefficient (ICC).

FC: functional connectivity, FCD: functional connectivity dynamics matrix, KS: Kolmogorov-Smirnov distance.

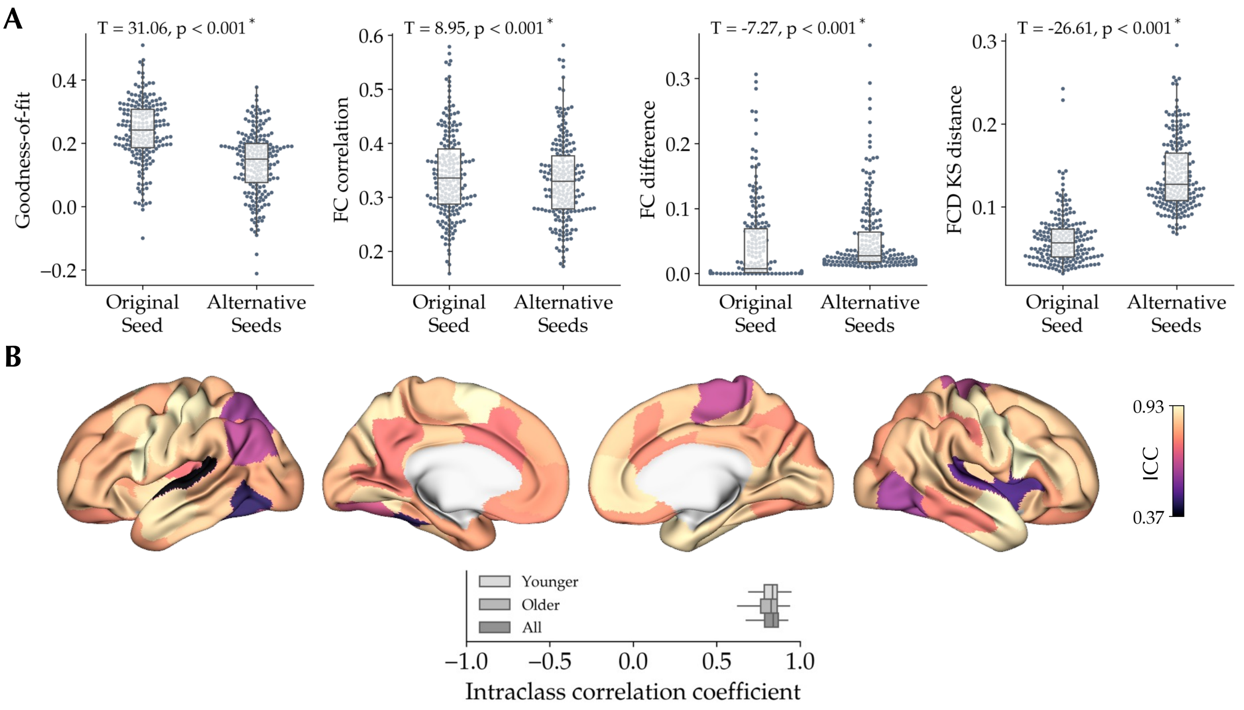

Fig. S16. Effects of varying Gaussian noise seed in the optimal simulations of the main run. (A) Comparison of goodness-of-fit measures of optimal simulation of the main run when using the default Gaussian noise seed and the median of 50 simulations (per subject) using alternative noise seeds, in the PNC subsample (N = 200). T- and p-values resulting from paired T-tests are reported. **(B)** Median of node-wise median absolute deviation intraclass correlation coefficient (ICC) of E-I ratio between the original simulation and the 50 simulations using alternative Gaussian noise seeds, calculated across all subsample subjects (dark gray; also shown on the map), older half (medium gray), or younger half (light gray). Paired T-test comparing ICCs of younger and older halves across nodes showed no significant difference.

FCD: functional connectivity dynamics matrix, KS: Kolmogorov-Smirnov distance.

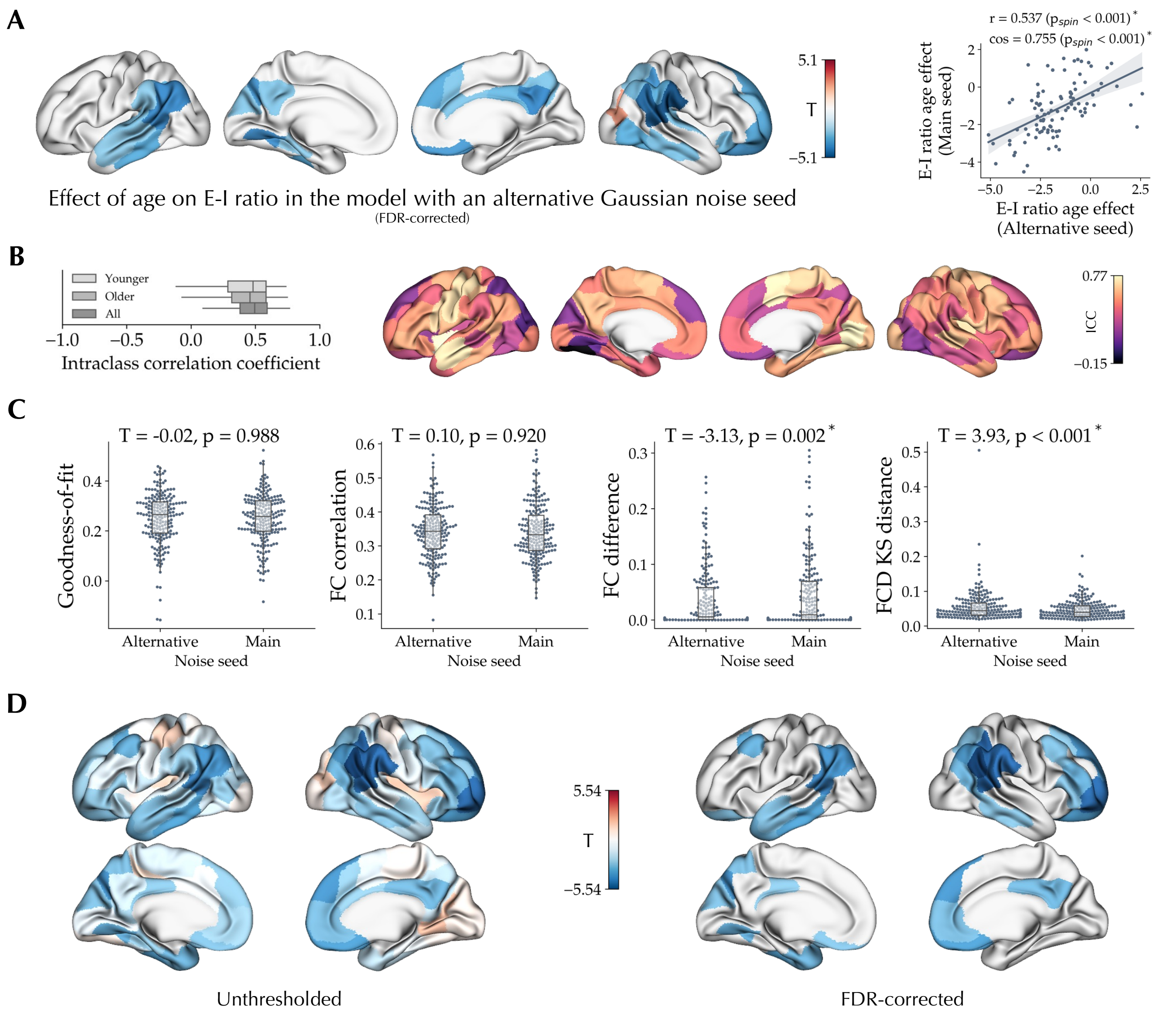

Fig. S17. Robustness of the findings when fitting the models using an alternative Gaussian noise seed. (A) *Left*: Effect of age on E-I ratio in the PNC subsample (N = 200) when the model simulation-optimization was run using an alternative Gaussian noise seed different from the main model, after false discovery rate (FDR) correction. *Right:* Spatial co-alignment (Pearson correlation [r] or cosine similarity [cos]) of the unthresholded effect of age on E-I ratio between models with the main versus alternative Gaussian noise seeds. **(B)** Distribution and map of the node-wise median absolute deviation intraclass correlation coefficient (ICC) of E-I ratio between the models with the main versus alternative Gaussian noise seeds, calculated across all subsample subjects (dark gray; also shown on the map), older half (medium gray), or younger half (light gray). Paired T-test comparing ICCs of younger and older halves across nodes showed no significant difference. **(C)** Comparison of goodness-of-fit measures between models fit with the main versus alternative Gaussian noise seeds. T- and p-values resulting from paired T-tests are reported. **(D)** Effect of age on E-I ratio in the PNC subsample (N = 200) based on averaged data of the models run using the main and alternative noise seeds.
FC: functional connectivity, FCD: functional connectivity dynamics matrix, KS: Kolmogorov-Smirnov distance.

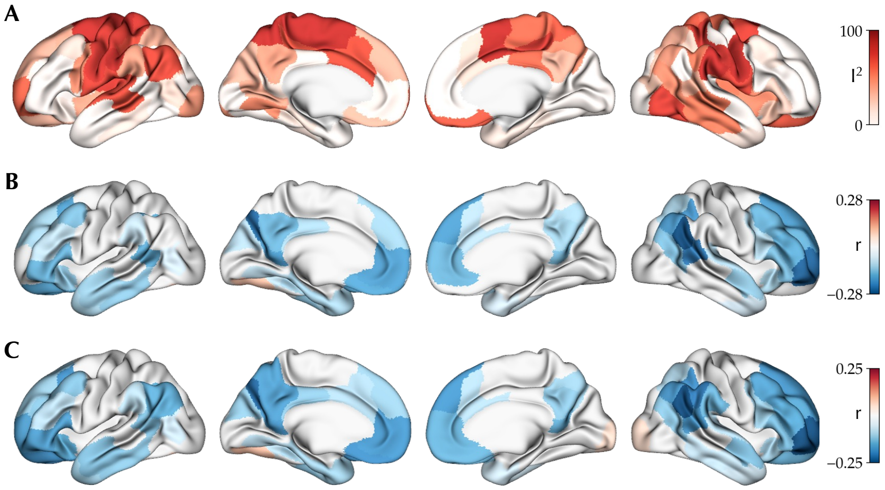

Fig. S18. Supplementary results of random-effects meta-analyses on the findings across alternative modeling configurations. (A) I^2 index indicating heterogeneity of the effect sizes (r, partial correlation of age with E-I ratio controlling for goodness-of-fit, sex and in-scanner rs-fMRI motion) across modeling configurations included in the meta-analysis (Fig. 5A-F) is shown for each parcel. Higher values indicate higher heterogeneity of the effects across alternative modeling configurations. **(B)** Significant (false discovery rate [FDR]-corrected) pooled partial correlation of age with E-I ratio across modeling configurations, in parcels with non-significant ($p(Q) > 0.05$) heterogeneity of the effect sizes. **(C)** Significant (FDR-corrected) pooled partial correlation of age with E-I ratio in random-effects meta-analyses in which results of both map-based (Fig. 5A-F) and map-free (Fig. S11D) models are included.

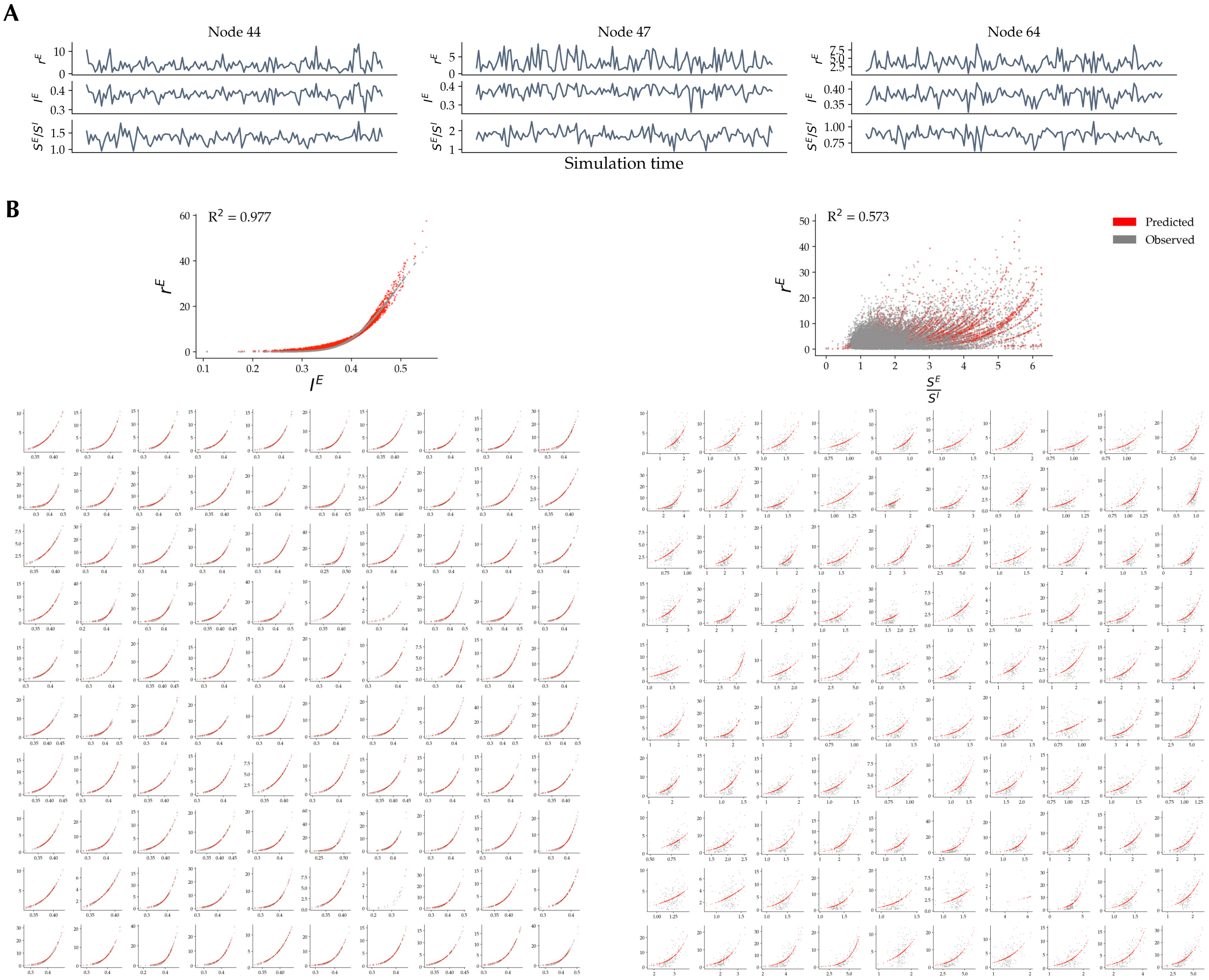

Fig. S19. Association of the r^E, I^E and S^E/S^I time series in the optimal simulation of an example subject. (A) Time series of r^E, I^E and S^E/S^I in three randomly selected nodes. **(B)** *Top*: Association of r^E with I^E (*left*) and S^E/S^I (*right*) across all nodes and time points based on a mixed generalized linear model with an exponential fitting function and random intercepts and slopes per node. Mixed generalized linear model prediction (red) and the observed simulation data (gray) are shown. Note that given S^E/S^I is a ratio and approaches towards infinity in some data points, we excluded the data points at the top 2.5 percentile of S^E/S^I. *Bottom*: Model prediction (red) and observed data (gray) for all 100 nodes are shown.

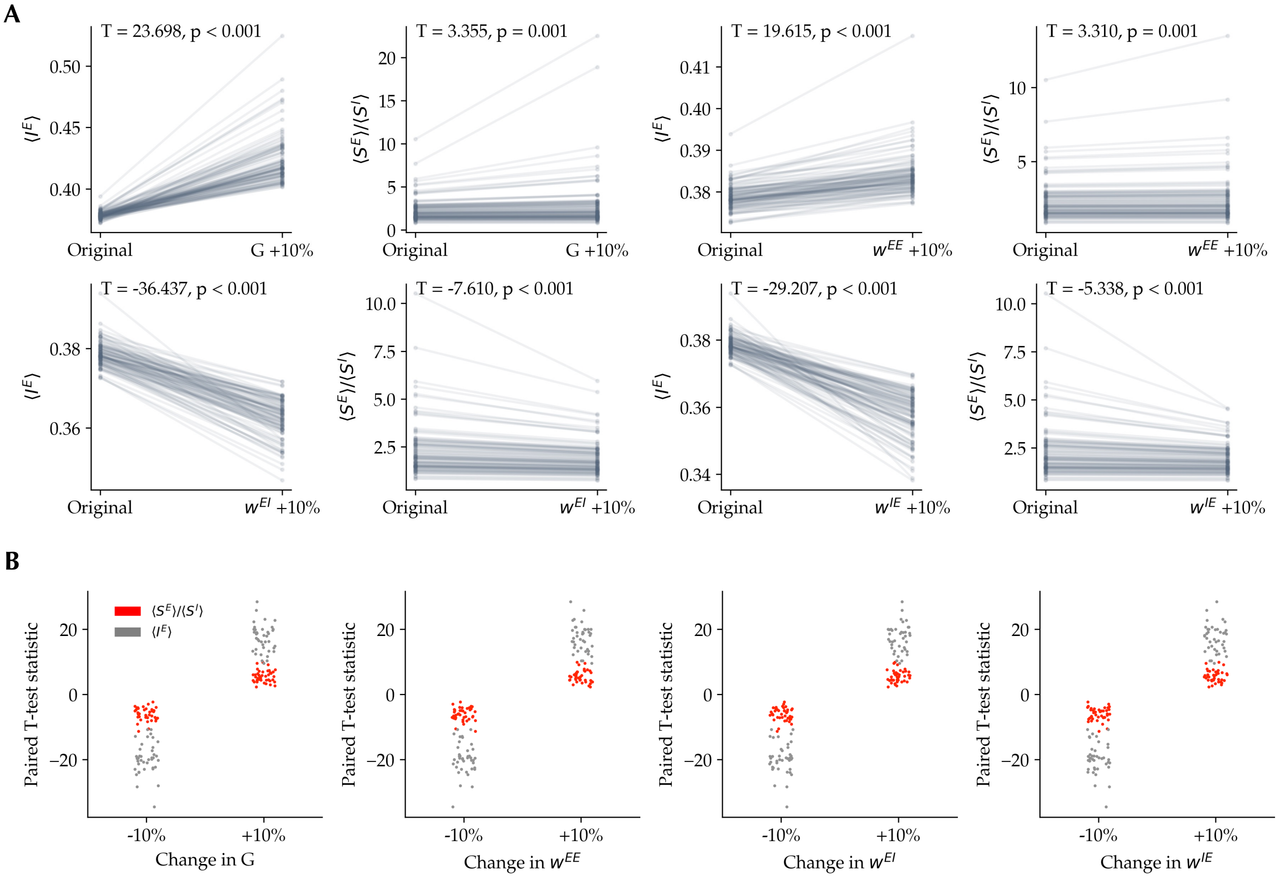

Fig. S20. Effect of model parameter perturbations on $\langle I_i^E \rangle$ and $\langle S_i^E \rangle / \langle S_i^I \rangle$. (A) Effect of 10% increase in each of the parameters on $\langle I_i^E \rangle$ and $\langle S_i^E \rangle / \langle S_i^I \rangle$ based on the optimal simulation of an example subject ('original' simulation). Paired T-test was used to compare $\langle I_i^E \rangle$ and $\langle S_i^E \rangle / \langle S_i^I \rangle$ across nodes between 'original' and 'perturbed' simulations. **(B)** Paired T statistics comparing $\langle I_i^E \rangle$ (gray) and $\langle S_i^E \rangle / \langle S_i^I \rangle$ (red) between 'original' and 'perturbed' simulations for 40 randomly selected subjects are shown in response to a 10% increase or decrease of each parameter.

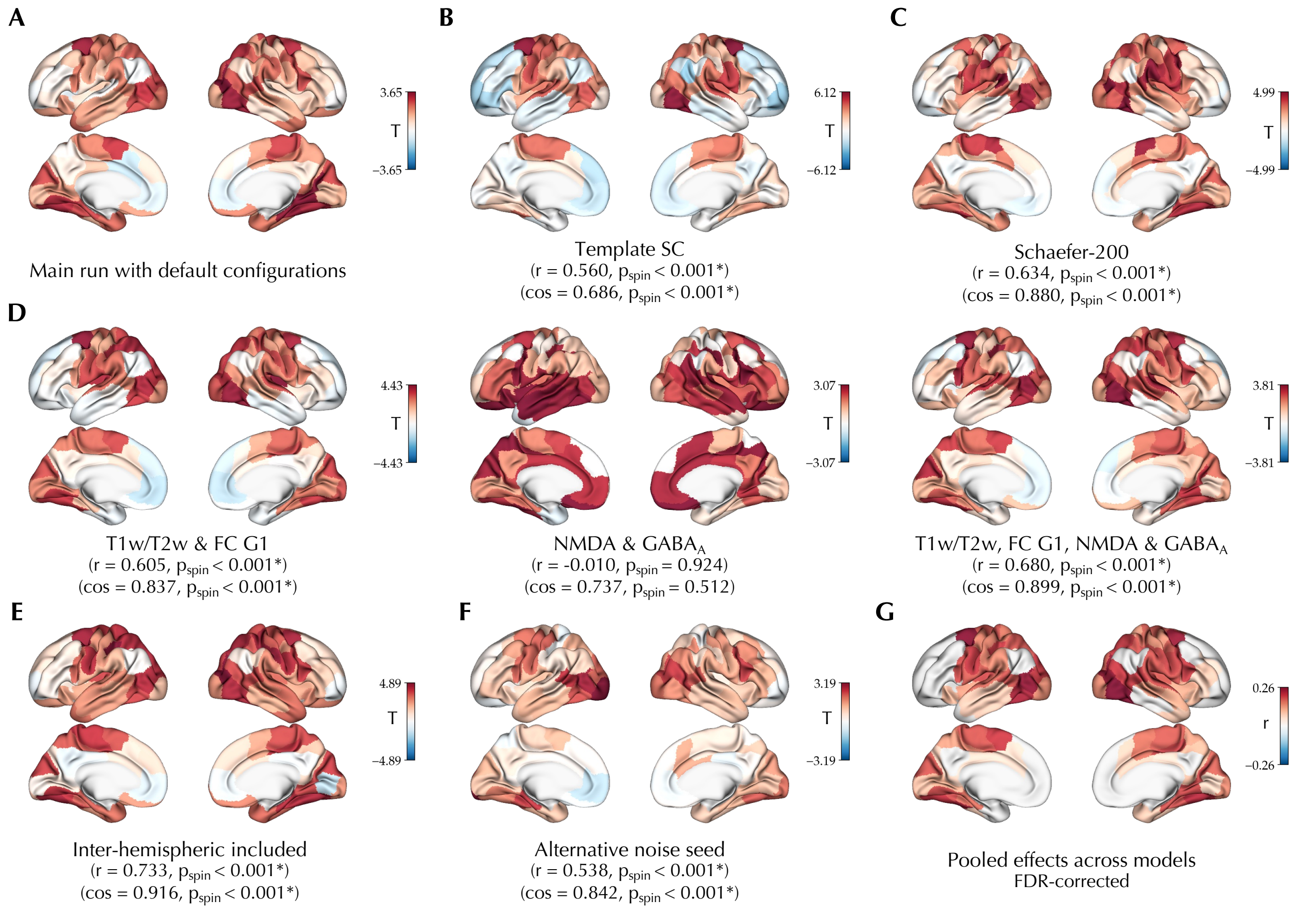

Fig. S21. Effect of age on $\langle S_i^E \rangle / \langle S_i^I \rangle$ and its robustness across different configurations in the PNC subsample. The unthresholded effect of age on $\langle S_i^E \rangle / \langle S_i^I \rangle$ observed in a random subsample of the PNC dataset (N = 200) using the default configurations (**A**) compared to age effects observed using alternative configurations, including: (**B**) using a fixed template SC based on the MICs dataset, (**C**) definition of nodes based on a Schaefer parcellation with a higher granularity of 200 nodes, (**D**) using alternative subsets of biological maps to determine the heterogeneity of regional parameters, (**E**) including the inter-hemispheric connections in the goodness-of-fit, and (**F**) using an alternative Gaussian noise seed. In panels **B**-**F** the statistics indicate spatial co-alignment (Pearson correlation [r] or cosine similarity [cos]) of each map with the $\langle S_i^E \rangle / \langle S_i^I \rangle$ age effect observed using the default configurations (Panel **A**). (**G**) Pooled partial correlation of age with $\langle S_i^E \rangle / \langle S_i^I \rangle$ (controlling for goodness-of-fit, sex and in-scanner rs-fMRI motion) across panels **A**-**F** based on random-effects meta-analyses.

T1w/T2w: T1-weighted to T2-weighted ratio, FC G1: principal gradient of functional connectivity, NMDA: N-methyl-D-aspartate receptor density, $GABA_{A/BZ}$: γ-aminobutyric acid type A/Bz receptor density.

Table S1. Sources for the multimodal maps of the sensorimotor-association cortical axis.

Map	Reference
Sensorimotor-association axis	Sydnor et al. (18)
T1-weighted to T2-weighted ratio	Glasser et al. (68, 69)
Principal gradient of functional connectivity	Margulies et al. (72)
Evolutionary expansion	Hill et al. (130)
Allometric scaling	Reardon et al. (131)
Oxygen cerebral metabolic rate	Vaishnavi et al. (132)
Glucose cerebral metabolic rate	Vaishnavi et al. (132)
Cerebral blood flow	Satterthwaite et al. (133)
Principal component of gene expression data	Hawrylycz et al. (71), Markello et al. (73)
Principal component of NeuroSynth	Yarkoni et al. (134), Poldrack et al. (135)
Principal gradient of laminar thickness covariance[a]	Saberi et al. (136)
Cortical thickness	Glasser et al. (68, 69)

[a] This map was obtained from Saberi et al. (136) while other maps were obtained from neuromaps package (74).
